# Cas9‐mediated mutagenesis of potato starch‐branching enzymes generates a range of tuber starch phenotypes

**DOI:** 10.1111/pbi.13137

**Published:** 2019-05-14

**Authors:** Aytug Tuncel, Kendall R. Corbin, Jennifer Ahn‐Jarvis, Suzanne Harris, Erica Hawkins, Mark A. Smedley, Wendy Harwood, Frederick J. Warren, Nicola J. Patron, Alison M. Smith

**Affiliations:** ^1^ John Innes Centre Norwich Research Park Norwich UK; ^2^ Quadram Institute Bioscience Norwich Research Park Norwich UK; ^3^ Earlham Institute Norwich Research Park Norwich UK

**Keywords:** starch‐branching enzyme, Cas9‐mediated mutagenesis, potato tuber, starch granule, starch structure

## Abstract

We investigated whether Cas9‐mediated mutagenesis of starch‐branching enzymes (SBEs) in tetraploid potatoes could generate tuber starches with a range of distinct properties. Constructs containing the Cas9 gene and sgRNAs targeting *SBE1*,*SBE2* or both genes were introduced by *Agrobacterium*‐mediated transformation or by PEG‐mediated delivery into protoplasts. Outcomes included lines with mutations in all or only some of the homoeoalleles of *SBE* genes and lines in which homoeoalleles carried several different mutations. DNA delivery into protoplasts resulted in mutants with no detectable Cas9 gene, suggesting the absence of foreign DNA. Selected mutants with starch granule abnormalities had reductions in tuber SBE1 and/or SBE2 protein that were broadly in line with expectations from genotype analysis. Strong reduction in both SBE isoforms created an extreme starch phenotype, as reported previously for low‐SBE potato tubers. HPLC‐SEC and ^1^H NMR revealed a decrease in short amylopectin chains, an increase in long chains and a large reduction in branching frequency relative to wild‐type starch. Mutants with strong reductions in SBE2 protein alone had near‐normal amylopectin chain‐length distributions and only small reductions in branching frequency. However, starch granule initiation was enormously increased: cells contained many granules of <4 μm and granules with multiple hila. Thus, large reductions in both SBEs reduce amylopectin branching during granule growth, whereas reduction in SBE2 alone primarily affects numbers of starch granule initiations. Our results demonstrate that Cas9‐mediated mutagenesis of *SBE* genes has the potential to generate new, potentially valuable starch properties without integration of foreign DNA into the genome.

## Introduction

Starch in potato (*Solanum tuberosum* L.) tubers has been the target of multiple types of genetic modification, for a variety of purposes. Earlier modifications were aimed at modification of starch properties for direct industrial use (e.g. Jobling, 2004; Jobling *et al*., 2002; Xu *et al*., 2014). Recent interest has focussed on altering starch properties to reduce the potentially deleterious effects of consuming starch on individuals prone to obesity and related conditions including type II diabetes (Birt *et al*., 2013). Potato starch is highly digestible when cooked (gelatinized). Rapid hydrolysis of gelatinized starch in the upper gut causes large increases in blood glucose, which in combination with other factors can result in poor insulin control of blood sugar levels and obesity. Consumption of potatoes with less digestible starch (so‐called resistant starch) would be expected to ameliorate these effects and might also have health benefits by providing substrates for fermentation by the lower gut microbiome (El Kaoutari *et al*., 2013; Keenan *et al*., 2015; Maier *et al*., 2017; Raigond *et al*., 2015). Microbiome fermentation produces short‐chain fatty acids (SCFA) with beneficial effects including maintenance of optimal function of pancreatic beta‐cells that produce insulin (Keenan *et al*., 2015; Pingitore *et al*., 2017).

Attempts to engineer resistant starches *in planta* have focussed on increasing the apparent amylose content of starch. Whereas the main component (about 70%) of starch, amylopectin, consists of α‐1,4 linked glucoses with relatively frequent, clustered branch points formed by α‐1,6 linkages, amylose is a largely linear α‐1,4 linked glucose polymer. The amylose content of starch profoundly influences its functional properties (Jobling, 2004). During gelatinization and cooling, long linear chains form double helices that pack together into crystallites. These are resistant to attack by α‐amylases in the upper gut. Starches with a high content of amylose (and/or long unbranched amylopectin chains) are generally more resistant to digestion than starches with low contents of amylose and other long, linear chains.

The main approach to increasing the amylose content of starch has been to reduce the activity of starch‐branching enzymes (SBEs), which introduce α‐1,6‐linkages into starch. A naturally occurring high‐amylose, digestion‐resistant starch in maize arises because of a mutation (*amylose‐extender* or *ae*) that eliminates a major form of SBE in the endosperm (Li *et al*., 2008). This phenotype has been reproduced in wheat and rice by transgenic and conventional means (e.g. Botticella *et al*., 2018; Butardo *et al*., 2011; Hazard *et al*., 2012; Regina *et al*., 2006, 2015; Sestili *et al*., 2015; Slade *et al*., 2012; Yano *et al*., 1985), and through application of CRISPR/Cas9‐mediated mutagenesis in rice (Sun *et al*., 2017). However, the loss of SBE isoforms can reduce grain starch content, yield and loaf volume in wheat (Schönhofen *et al*., 2017) and grain weight in rice (Sun *et al*., 2017). The commercial viability of such mutants is unclear.

Very large reductions in SBE activity in potato tubers result in high‐amylose starch with profoundly altered properties (Blennow *et al*., 2005; Schwall *et al*., 2000) and decreased digestibility (Karlsson *et al*., 2007; Zhao *et al*., 2018). Essentially identical results were achieved by three different means of reducing expression of both SBE isoforms (SBE1 and SBE2): antisense RNA (Schwall *et al*., 2000), RNAi (Andersson *et al*., 2006) and expression of single‐domain SBE‐specific camelid antibodies (Jobling *et al*., 2003). Tuber starch from some transformed lines had apparent amylose contents >50% (compared with about 30% in wild‐type tubers), and deeply cracked and fissured starch granules that failed to gelatinize fully even when boiled (normal gelatinization temperature is ~60 °C). The transformed plants also differed from wild‐type plants in several ways likely to affect their commercial viability. These included tuber size and shape, starch, water and sugar content, cooking properties, and whole‐plant growth and nitrogen economy (Hofvander *et al*., 2004; Karlsson *et al*., 2007; Pourazari *et al*., 2018). The transgenic nature of the high‐amylose tubers also made them unacceptable for nutrition studies in Europe.

Our aim was to investigate Cas9‐mediated targeted mutagenesis as a means of reducing the capacity for starch branching in potato tubers. We wished to discover whether this technology can produce transgene‐free potatoes with a range of levels of amylose and/or long amylopectin chains, as a basis for future development of plants that have reduced starch digestibility without the deleterious secondary effects seen in plants with very high apparent amylose contents. We envisaged that such lines could be generated by targeted mutations designed to reduce and/or modify SBE protein and activity without eliminating it. As described above, near elimination of both isoforms has drastic effects on starch. Near elimination of SBE1 alone has little effect on starch structure (Safford *et al*., 1998), and near elimination of SBE2 alone results in relatively small elevations of amylose content (Jobling *et al*., 1999).

To assess whether Cas9‐mediated targeted mutagenesis could be used to generate a range of starch composition and structure, we assembled constructs designed to induce mutations in *SBE1* and *SBE2*, either individually or together, in the tetraploid potato cultivar Desiree. We generated plants with mutations in *SBE* genes by both *Agrobacterium*‐mediated transformation and PEG‐mediated DNA delivery into protoplasts. Recent research shows that mutated potato plants free of foreign DNA can be recovered from transformed protoplasts transiently expressing CRISPR/Cas9 constructs (Andersson *et al*., 2017). Introduction of mutations without integration of foreign DNA is desirable because removal of a transgene by crossing and segregation in a tetraploid potato would result in loss of the variety (Stokstad, 2019). The use of these two approaches to introduce constructs containing Cas9 and single guide RNAs (sgRNAs) produced a wide range of mutations in the *SBE* genes and resulted in diverse starch phenotypes.

## Results

### The *Solanum tuberosum* genome encodes three isoforms of SBE

Coding sequences of two potato SBE isoforms have previously been cloned (Jobling *et al*., 1999; Khoshnoodi *et al*., 1996). Analysis of the reference potato genome (*S. tuberosum* Group Phureja clone DM1‐3 516R44; The Potato Genome Sequencing Consortium, 2011) revealed that SBE1 is coded across 15 exons on chromosome 4 while SBE2 is coded across 22 exons on chromosome 9 (Figure S1). A gene encoding a third SBE‐like protein was also identified. It belongs to a distinct clade of SBE‐like proteins found previously in potato (Van Harsselaar *et al*., 2017) and in species including Arabidopsis, *Populus*, rice and maize (Dumez *et al*., 2006; Han *et al*., 2007; Wang *et al*., 2010, 2014; Yan *et al*., 2009). The potato SBE‐like protein has 39% and 37% identity at the amino acid level to SBE1 and SBE2, respectively. In Arabidopsis, null mutations in the orthologous gene (At3g20440, designated *BE1* by Dumez *et al*., 2006) are embryo‐lethal (Wang *et al*., 2010) and plants carrying a weak mutant allele are defective in organogenesis from calli (Wang *et al*., 2014). It is unlikely that this protein is directly involved in starch synthesis (Wang *et al*., 2010), and it was not targeted for mutagenesis.

Analysis of the coding sequences amplified and cloned from RNA isolated from the tetraploid potato Desiree identified two copies of both *SBE1* (designated *SBE1.1* and *SBE1.2*) and *SBE2* (designated *SBE2.1* and *SBE2.2*), likely to be homoeologues (Figure S2). At the level of predicted amino acid sequence, SBE1 and SBE2 homoeologues were 97% and 99% identical, respectively (881/905 amino acids identical for SBE1.1 and 1.2; 874/878 amino acids identical for SBE2.1 and 2.2).

### *Agrobacterium*‐mediated stem transformation yielded plants with a range of different mutations

Target sequences for Cas9‐mediated mutagenesis of *SBE1* and *SBE2* were selected from the genome sequence (Figure S1), and their conservation in cv Desiree was checked. Although there were minor differences between the sequences of the Phureja and Desiree *SBE* genes, the selected target sequences were identical in the two cultivars. Four sgRNAs were designed for *SBE1*, recognizing targets in exon 4 (sgRNA1), exon 5 (sgRNA2) and exon 6 (sgRNAs 3 and 4; Figure S1). Six sgRNAs were designed for *SBE2*, recognizing targets in exon 7 (sgRNA5), exon 8 (sgRNA6), exon 12 (sgRNA7), exon 13 (sgRNA8) and exon 14 (sgRNAs 9 and 10; Figure S1).

Constructs for *Agrobacterium*‐mediated transformation contained a selection cassette, the Cas9 cassette, and either two or four sgRNA expression cassettes (Figure S3). Two constructs containing different combinations of sgRNAs were designed for each of *SBE1*,* SBE2* and both *SBE*s (Figure S3). The six constructs were introduced separately into stem explants using *Agrobacterium*‐mediated transformation, and regenerated plantlets were recovered from calli on selection medium. Using primers for the Cas9 gene, the presence of the integrated transgene was confirmed in all plantlets tested. Plantlets were screened by PCR amplification of the target loci from genomic DNA (gDNA) extracted from leaves. In the first instance, mutations were identified by a reduction in amplicon size observed by agarose gel electrophoresis, indicating deletions between two sgRNA targets (Figures S4 and S5A). These lines were further characterized by direct sequencing or by cloning and sequencing of the amplicon. All six constructs resulted in plantlets with mutations in the target genes. In total, 767 plantlets were recovered from 374 calli (usually two or three from each callus). Of these, we detected 45 plantlets (6%) with mutations at the targets. Fourteen had mutations in *SBE1*, 24 had mutations in *SBE2,* and 7 had mutations in both *SBE1* and *SBE2* (Table S1). Our screen would not have identified plants in which *SBE* genes contained only small mutations, so the numbers of plants carrying mutations may be underestimated.

**Figure 1:**
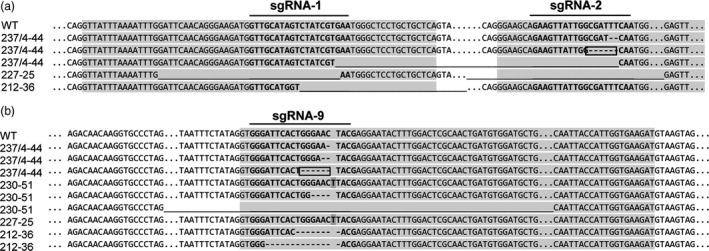
Sequence alignments showing mutations in a, *SBE1* and b, *SBE2* in four plant lines. Exons are shaded in grey, and target sequences of sgRNAs 1, 2 and 9 are shown in bold letters. Long stretches of sequence are indicated by dots (‘…’); short nucleotide deletions by dashes, ‘‐’; longer deletions by continuous lines, ‘_’ and; single nucleotide insertions by dark shading. In‐frame deletions are boxed.

Each construct gave rise to several different types of mutation, including large and small deletions and insertions (Figure 1). Constructs targeting both *SBE* genes resulted in mutations in both genes in some cases, but in only one or the other gene in other cases. For a given callus, independent plantlets frequently carried the same mutations, but in some instances, calli gave rise to both mutated and nonmutated plantlets, or plantlets carrying different mutations.

### PEG‐mediated protoplast transformation resulted in mutated plants with no Cas9 gene

Constructs for PEG‐mediated delivery of DNA into protoplasts used the sgRNAs described above (Figure S3). For each construct, versions were made with or without the coding sequence for YFP inserted in frame after the Cas9 sequence. The presence of YFP allowed transformation efficiency to be assessed by fluorescence microscopy (see Figure S6). Leaf protoplasts were incubated with plasmid DNA in the presence of PEG and calcium and then allowed to regenerate without antibiotic selection. Controls contained water rather than plasmid DNA.

Regenerating calli were screened for the presence of mutations as described above for *Agrobacterium*‐mediated transformation (Table S1, Figures S4 and S5B). In total, 665 calli were screened, and mutations were found in ten. The low efficiency of mutagenesis likely reflects the lack of selection pressure for the presence of either the Cas9 gene or the mutations. Consistent with this explanation, there was no Cas9 transgene in plantlets regenerated from seven of the 10 calli (Figure S7). The absence of the transgene also indicates that mutations were induced following transient expression of the sgRNA/Cas9 ribonuclease complex from plasmid DNA.

### Lines with different mutations contained different amounts of SBE proteins

Starch was prepared from tubers of senescing glasshouse‐grown plants propagated from plantlets with mutations in *SBE* genes. Starch granules from four independent lines, in which either *SBE2* alone or both *SBE1* and *SBE2* were targeted, had pronounced alterations in granule appearance (described in the next section). We conducted detailed genotyping of the *SBE* genes and analysed the SBE protein content of these lines. None of the lines in which *SBE1* alone was targeted had altered starch granules.

**Figure 2:**
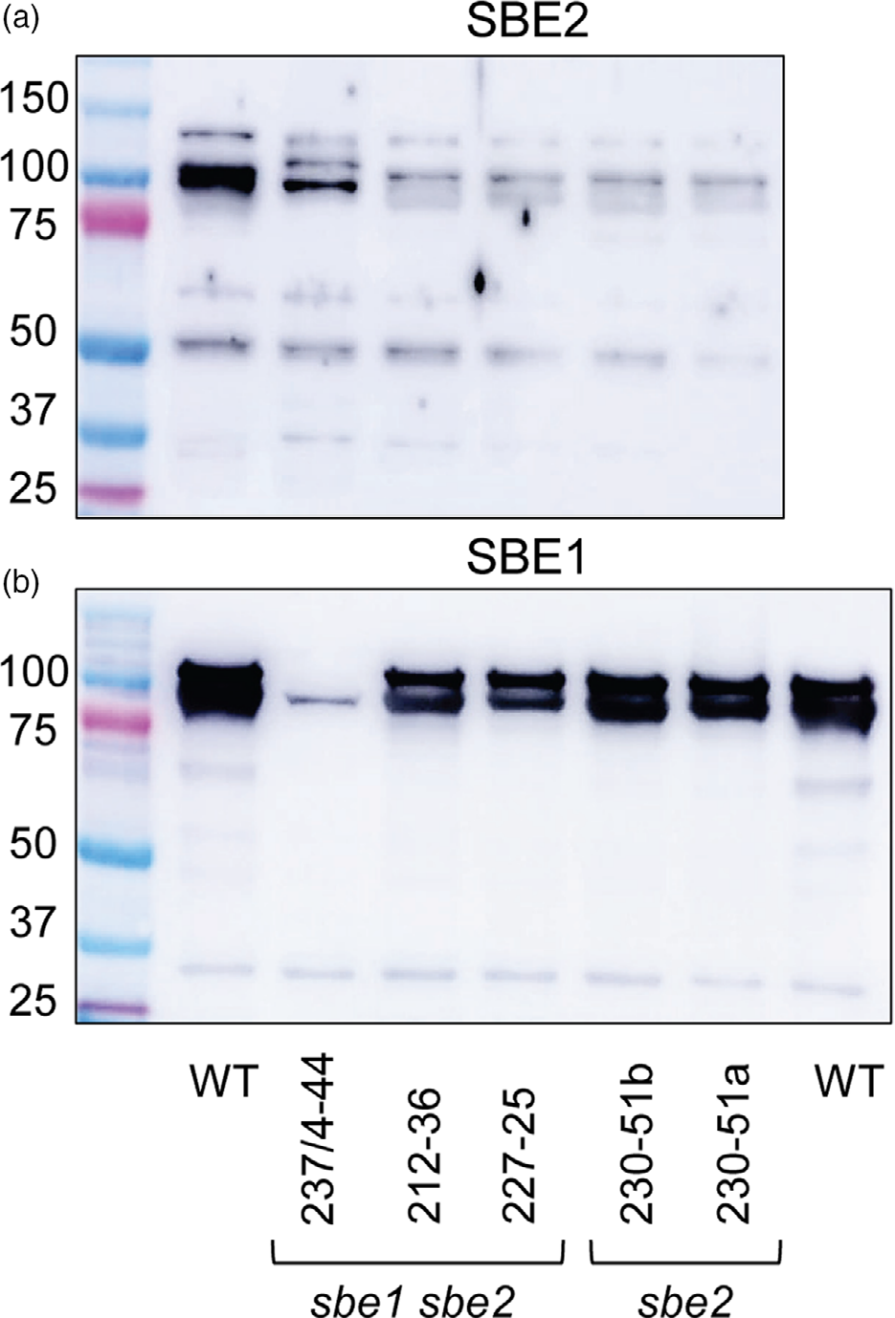
Occurrence of SBE1 and SBE2 proteins in extracts of tubers of wild‐type and mutant plants. The soluble fraction of extracts of mature tubers (samples from the mid‐cortical region half way along the tuber) were subjected to SDS‐PAGE on 7.5% acrylamide gels. Each lane contained 20 μg soluble protein. Gels were blotted onto PVDF membranes, probed with 1/1000 dilutions of SBE1 or SBE2 antiserum and developed using an anti‐rabbit antiserum and an enhanced chemiluminescence kit. Left lanes contain marker proteins; molecular masses are indicated in kDa. (a) Probed with SBE2 antiserum. (b) Probed with SBE1 antiserum. The occurrence of two bands of SBE1 protein has been observed previously (Jobling *et al*., 1999). Mutants 230‐51a and 230‐51b are derived from two independent shoots from the same callus and are genetically identical.

Line 237/4‐44 (*SBE1* and *SBE2* mutated) was obtained by protoplast‐mediated transformation with construct 237 (Figure S3). We found three different repair events in *SBE1*: a two base‐pair deletion resulting in a premature stop codon; a six base‐pair in‐frame deletion resulting in the loss of amino acids 169D and 170F; and a large (189 bp) deletion of the DNA between two sgRNAs (Figure 1 and Figure S8). Differences between the identified homologues of *SBE1* (Figure S2) downstream of the target site for sgRNA2 indicate that the 2 and 189 bp deletions occurred in *SBE1.1* while the 6 bp deletion occurred in *SBE 1.2* (Figure S8). These data suggest biallelic mutations of *SBE1.1* and homozygous mutation of SBE*1.2*. Analysis of *SBE2* was also consistent with three different repair events, two with deletions leading to premature stop codons and one leading to loss of amino acids 524G and 525N (Figure 1 and Figure S9). The existence of one allele each of *SBE1* and *SBE2* with small in‐frame deletions is consistent with immunoblot analysis of SBE proteins in the tubers: SBE1 and SBE2 proteins were reduced but not absent (Figure 2). The absence of the Cas9 transgene in this line (Figure S7) indicates that the mutations were induced by transient expression of the sgRNA‐Cas9 ribonuclease complexes in the protoplast and therefore that the line is unlikely to be genetically chimeric.

Line 230‐51 (*SBE2* mutated) was obtained by *Agrobacterium*‐mediated transformation with construct 230 (Figure S3). Analysis of *SBE2* indicated that all alleles were mutated and provided evidence of three different repair events including a large deletion and mutations leading to premature stop codons (Figure 1 and Figure S10). This is consistent with immunoblot analyses, which showed the loss of SBE2 protein and normal levels of SBE1 (Figure 2) in tubers. Two plantlets (230/51a and 230/51b) derived from the same callus carried the same mutations, suggesting that they arose early in callus formation and thus that the line is unlikely to be genetically chimeric.

Lines 227‐25 and 212‐36 (*SBE1* and *SBE2* mutated) were obtained by *Agrobacterium*‐mediated transformation with constructs 227 and 212, respectively (Figure S3). For both lines, there was evidence of both mutated and nonmutated sequences for *SBE1* (Figure 1, Figures S11 and S12). Consistent with this finding, amounts of SBE1 protein were somewhat reduced in both lines (Figure 2). No wild‐type *SBE2* was detected in 212‐36, where multiple repair events were detected (Figure 1 and Figure S13). This is consistent with the loss of SBE2 protein in tubers (Figure 2). Analysis of *SBE2* in line 227‐25 provided evidence of both mutated and wild‐type sequences (Figure 1 and Figure S14); however, SBE2 protein was not detected in tubers (Figure 2). These data do not exclude the possibility that lines 227‐25 and 212‐36 may be genetically chimeric, with different mutations in leaf cells from which gDNA was extracted and tuber cells from which protein was extracted.

### Mutations in *SBE2* or both *SBE* genes result in diverse starch phenotypes

**Figure 3:**
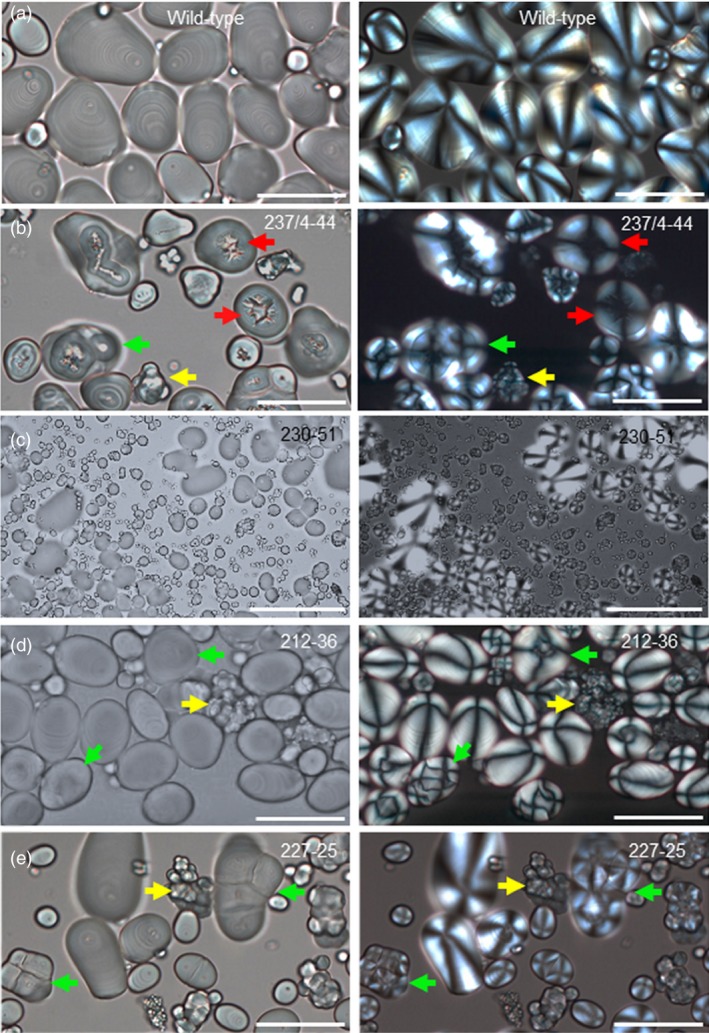
Starch granules from mutant and wild‐type plants viewed under normal (L) and polarized (R) light. (a) Wild‐type starch granules, (b) granules from mutant line 237/4‐44. (c) Granules from mutant line 230‐51, focussed to show huge numbers of tiny granules. (d) Granules from mutant line 212‐36. (e) Granules from mutant line 227‐25. Arrows indicate examples of cracking (red), nodular granules (yellow) and granules with multiple hila (green). Scale bars represent 50 μm.

**Figure 4:**
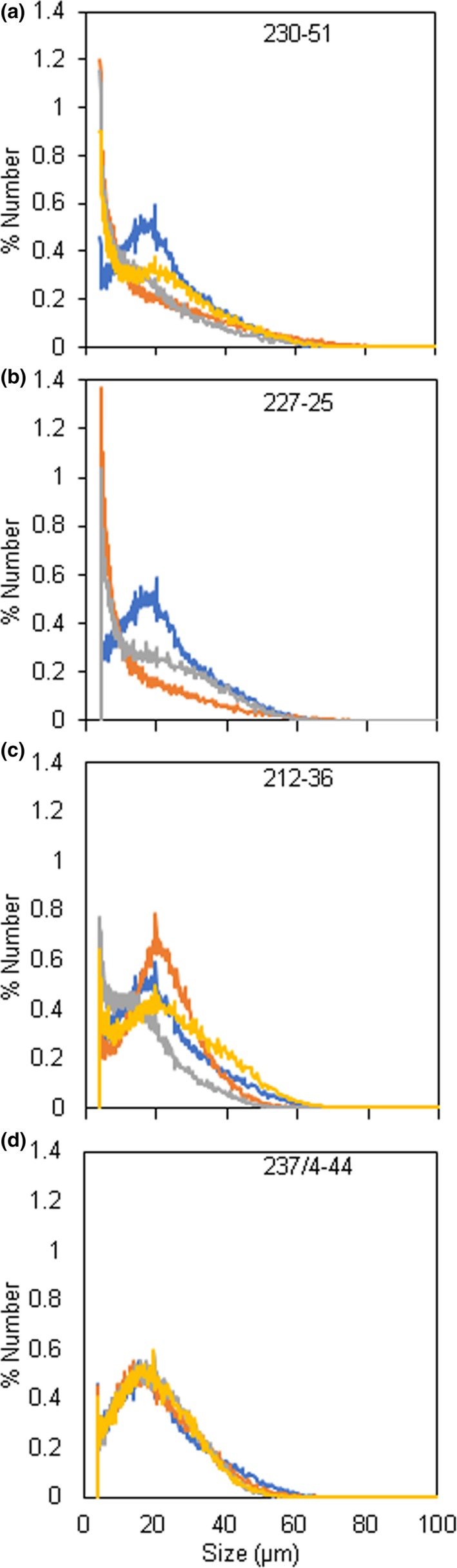
Distributions of starch granule sizes between 4 and 100 μm. Results were obtained from analysis of purified tuber starch using a Multisizer 4e Coulter counter. Graphs show the % of total granules (*y*‐axis) of a particular diameter (*x*‐axis). (a) Wild‐type starch (blue) and three independently prepared samples of starch (yellow, grey, orange) from mutant line 230‐51. (b) Samples as for a, but showing two independently prepared starch samples from mutant line 227‐25. (c) Samples as for a, but showing three independently prepared starch samples from mutant line 212‐36. (d) Samples as for a, but showing three independently prepared starch samples from mutant line 237/4‐44.

One of the four independent lines with distinctive starch granule phenotypes, line 237/44‐4, (*SBE1* and *SBE2* mutated) had extreme starch granule abnormalities, similar to those described previously for potato plants with strongly reduced SBE activity/protein (Andersson *et al*., 2006; Glaring *et al*., 2006; Hofvander *et al*., 2004; Jobling *et al*., 2003; Schwall *et al*., 2000). Many granules were deeply cracked and fissured at the centre. Under polarized light, a high proportion of granules exhibited a complex birefringence pattern consistent with several separate origins of growth (hila). (In wild‐type granules, which have a single hilum, polarized light reveals a single Maltese cross‐centred on the hilum.) Some granules in line 237/44‐4 were nodular and appeared to have arisen from fusion of multiple granules (Figure 3 and Figure S15). Tuber starch content was not statistically significantly different from that of wild‐type plants (Figure S16), and the use of a Coulter counter (particle sizer) did not reveal major differences in the granule size profile relative to wild‐type plants (Figure 4).

**Figure 5:**
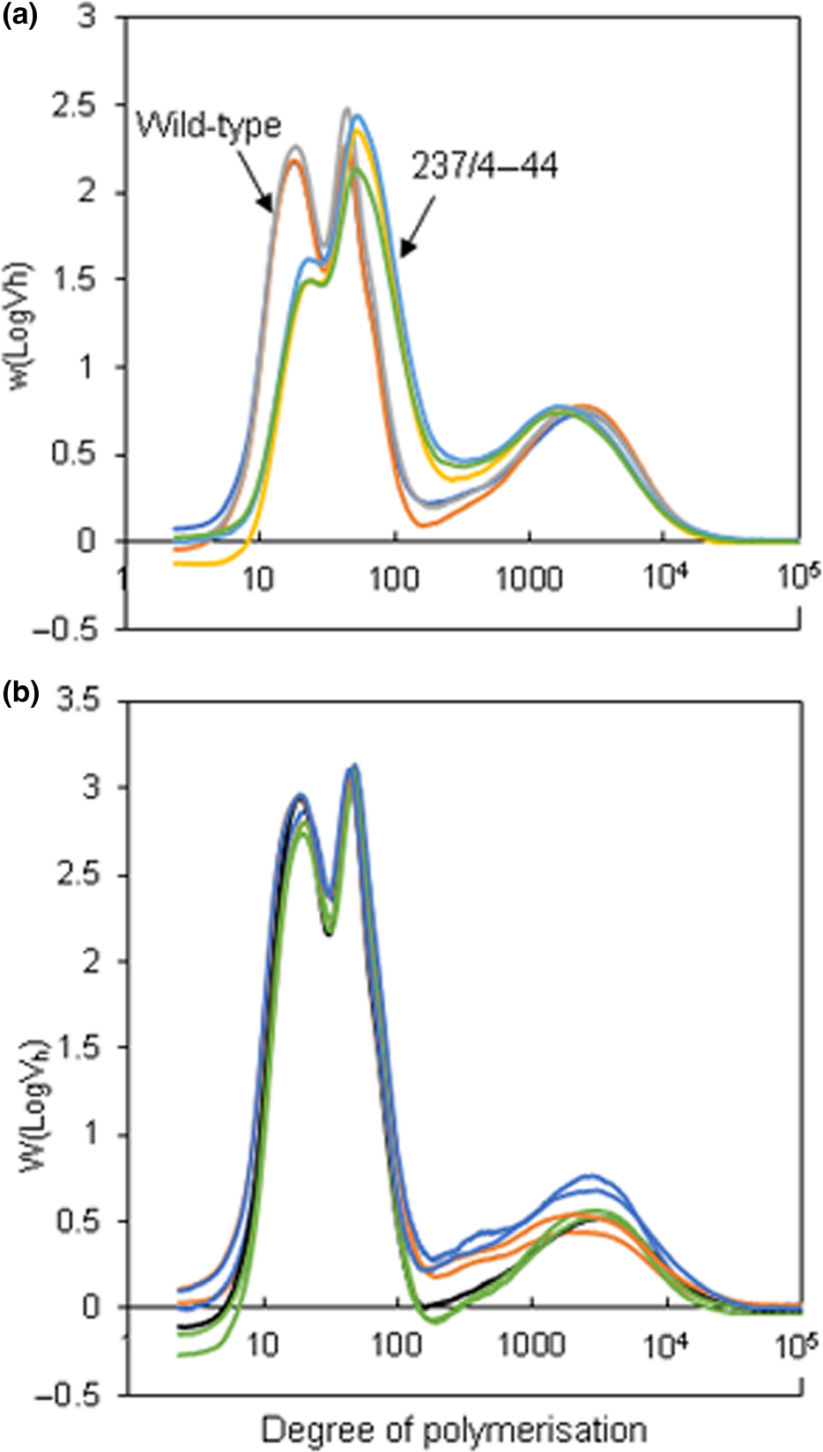
Chain‐length distributions of debranched starch. Purified starch from mature tubers was debranched with isoamylase and subjected to HPLC‐SEC. The *y*‐axis shows weight distribution, based on the relationship between elution volume and hydrodynamic radius (log*V*_h_) for pullulan standards. Note that the *x*‐axis is on a log scale. Each line represents an independently purified starch sample and is the mean of values from duplicates of that sample. (a) Starch from wild‐type (grey, dark blue and brown lines) and the *sbe1 sbe2* mutant 237/4‐44 (pale blue, yellow and green lines). (b) Starch from a control line (black line), sbe2 mutant 230‐51 (green lines), *sbe1 sbe2* mutant 212‐36 (orange lines) and *sbe1 sbe2* mutant 227‐25 (blue lines).

As expected from the severe granule phenotype, debranched starch from the 237/4‐44 mutant had a radically altered distribution of chain lengths relative to wild type. Information on the distribution of chain lengths was obtained from calibrated HPLC‐SEC outputs (Perez‐Moral *et al*., 2018; Wu *et al*., 2014), which provide information about weight distributions of chains as a function of hydrodynamic volume. This approach revealed three major peaks of chains in starch from control plants (protoplast‐derived wild‐type plants; Figure 5). The peak at dp 20 contains amylopectin chains that lie within a single crystalline lamella in the granule matrix, the peak at dp 50 contains long amylopectin chains that span crystalline lamellae, and the peak at dp 2500 contains amylose chains (Perez‐Moral *et al*., 2018). In the 237/4‐44 mutant, the short chains were strongly depleted, and the abundance and length of long amylopectin chains were increased (Figure 5a). The average length of amylose chains was also somewhat reduced: analysis of further lines will be required to confirm whether this is a consistent feature in plants with strongly reduced SBE. Further information about starch structure in the 237/4‐44 mutant was obtained by ^1^H NMR analysis of the relative frequencies of α‐1,4 and branch‐forming α‐1,6 linkages. The degree of branching (the number of branch points as a percentage of total glycosidic linkages) in 237/4‐44 starch was half that in wild‐type starch (Table 1).

**Table 1:** Degree of starch branching estimated from ^1^H NMR

Line	Mutated *SBE*	Batch 1	Batch 2
		Degree of branching (%)	
Wild‐type control (C)		2.22	n.d.
Wild‐type control (P)		2.11	2.11
237/4‐44a	*sbe1/sbe2*	1.12	n.d.
237/4‐44b		1.04	n.d.
212‐36	*sbe1/sbe2*	2.00	2.04
227‐25	*sbe1/sbe2*	1.83	1.89
230‐51a	*sbe2*	1.78	1.88
230‐51b		1.88	1.79

n.d., not done.

^1^H NMR spectra of solubilized starch were used to quantify α‐1,4 and α‐1,6 linkages, from which the % of linkages that are branching points was calculated. Wild‐type controls were untransformed plants (C) or plants regenerated from mock‐transformed protoplasts (P). Batch 1 and Batch 2 are analyses of independently purified batches of starch from one genotype. Lines 230‐51a and 230‐51b are two genetically identical plants from the same callus. Values are means of two technical replicates.

Starch granules from line 230‐51 (*SBE2* mutated), 227‐25 and 212‐36 (*SBE1* and *SBE2* mutated) had different defects from those in the extreme mutant. Some granules in these lines were normal in appearance, but a large fraction was not. Starch from these lines resembled that of the extreme line with respect to the occurrence of nodular granules and granules with multiple hila, but there were no cracks and fissures associated with hila (Figure 3). The most striking difference between these three lines (referred to as ‘intermediate lines’ below) and both the wild‐type and extreme lines was the presence of huge numbers of tiny granules. Although these tiny granules constituted a very small fraction of the total starch volume, the vast majority of granules in two out of three intermediate lines were <4 μm in diameter (Figure 4) and frequently around 1 μm or less (estimated from light microscopy) whereas the most frequent size class for wild‐type granules was 17–19 μm in diameter. Starch from line 212‐36 showed differences in particle size distributions between individual tubers. Although tiny granules were present, particles of about 20 μm diameter were also very abundant. We attribute this variation to high levels of clumped granules—apparently formed of numerous tiny granules adhering together—in this line. It seems likely that the extent to which the clumps dispersed during starch preparation varied between preparations.

**Figure 6:**
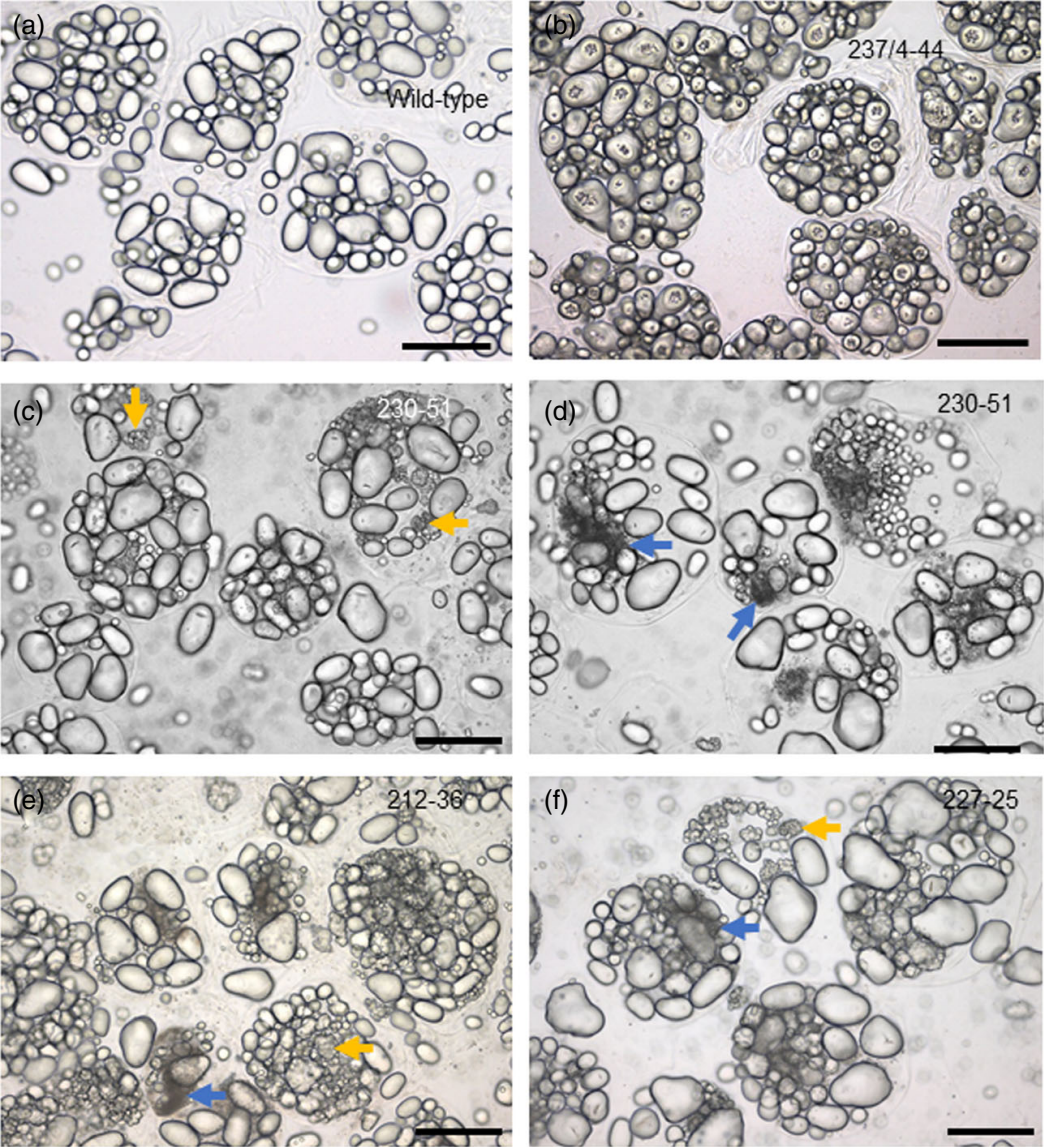
Starch granules within isolated tuber cells. Tuber slices were incubated with 50 mm CDTA for 3 days; then, cells were separated by gentle pressure. (a) Wild type. (b) Mutant line 237/4‐44. (c, d) Mutant line 230‐51. (e) Mutant line 212‐36. (f) Mutant line 227‐25. Arrows illustrate examples of nodular granules (yellow) and clusters of tiny granules (blue). Scale bars represent 50 μm.

We investigated whether essentially normal, abnormal and tiny granules were in the same or different cells. Incubation of fixed tissue with CDTA facilitated separation of tuber cells. For several different tubers, every cell examined contained both granules that were normal in appearance and granules that were nodular or had multiple hila. The ratios of apparently normal granules, nodular granules, granules with multiple hila and tiny granules also varied greatly from one cell to the next. Tiny granules were clustered in discrete regions, seen as dark patches in light‐microscope images (Figure 6 and Figure S15a,b). These data suggest that the initiation and growth of granules within a single cell differed profoundly from one amyloplast to another.

Despite the abnormal appearance and size profile of starch granules, HPLC‐SEC chain‐length profiles for starch from the intermediate lines were generally similar to those of wild‐type plants (Figure 5B). Both of the *sbe1 sbe2* mutants had somewhat higher levels of long amylopectin chains than wild‐type whereas the *sbe2* mutant did not: analysis of further lines will be required to determine whether this is a consistent difference between *sbe1 sbe2* lines and *sbe2* lines. ^1^H NMR analysis indicated that the frequency of branch points in starch from these mutants may be slightly reduced relative to wild‐type starch (Table 1). These mutants were estimated to have 85%–95% of the wild‐type degree of branching.

## Discussion

### Cas9‐mediated mutagenesis produced a wealth of different mutations

Our results show that Cas9‐mediated mutagenesis of *SBE* genes in potato can generate a great diversity of mutations. For each construct, we recovered plants with mutations of different sizes. The extent to which *SBE* genes were mutated also varied considerably. In some plants, wild‐type sequence was detectable while in others transformed with the same construct all targets were mutated. This kind of variability has been observed previously for Cas9‐mediated mutagenesis of potatoes (Andersson *et al*., 2017; Kusano *et al*., 2018; Nakayasu *et al*., 2018).

Our data suggest two possibilities for the presence of both wild‐type and mutated *SBE* sequences in some lines. First, some of the four alleles for each gene may have escaped mutagenesis. Andersson *et al*. (2017) provided a detailed analysis of the extent of mutation at a gRNA target site in the potato granule‐bound starch synthase gene (*GBSS*). They showed that—depending on the experimental conditions—up to 67% of plants recovered for a given sgRNA were mutated in multiple alleles, but only a very small percentage was mutated in all four alleles. Second, it is possible that some of our plants were genetic chimeras, containing both mutated and wild‐type cells. At least some of the calli that produced mutated shoots were chimeras: they gave rise to both mutated and unmutated plantlets, and—for calli regenerated from protoplasts—plantlets that had or did not have the Cas9 gene. For other lines, both sequencing data and the presence of abnormal starch granules in all tuber cells examined suggest that the genotypes were uniform and stable across the plant.

Results of immunoblotting confirmed that SBE protein levels were in most cases broadly in line with expectations from the analysis of the mutated genes. For example, in one line (237/4‐44), one *SBE1* and one *SBE2* allele contained only small in‐frame deletions, resulting as expected in some residual SBE1 and SBE2 proteins. In other lines, there were no wild‐type alleles and no protein. It is not known whether homoeoalleles of *SBE* genes in potato are equally expressed and make equal contributions to protein levels in the plant. If their contributions are not equal, the extent to which a given mutation affects protein levels may depend upon whether it is in a strongly expressed homoeoallele or a weakly expressed homoeoallele. Allelic variation in gene expression has been reported frequently for hybrids and polyploids in several species (e.g. Guo *et al*., 2004; Harper *et al*., 2016; Zhuang and Adams, 2007).

It is technically extremely difficult to determine the extent to which the mutations affected the capacity for starch branching in tuber cells, because the assayable activity of SBE1 and SBE2 does not reflect their relative importance in starch branching. Antisense‐mediated reduction in SBE1 reduced total SBE activity in tuber extracts by up to 98% without any effect on chain‐length profiles below dp70 or on iodine‐based measures of amylose content (Safford *et al*., 1998). By contrast, antisense‐mediated reduction in SBE2 was reported to have no effect on activity in tuber extracts but readily detectable effects on chain‐length distribution and iodine‐based measures of amylose content (Jobling *et al*., 1999; Schwall *et al*., 2000). These data imply that the assayable activity in tubers is largely attributable to SBE1 and is not a measure of the capacity for branching *in vivo*.

### Extreme phenotypes produced by Cas9‐mediated mutation of SBEs resembled those produced by antisense and RNAi approaches

The most extreme starch phenotypes we observed are similar to those in previous studies in which SBE protein in tubers was severely reduced by antisense or RNAi technology or the expression of neutralizing antibodies (Hofvander *et al*., 2004; Jobling *et al*., 2003; Schwall *et al*., 2000). Our data show that these phenotypes occur only when there is a strong reduction in both SBE2 and SBE1 proteins. Loss of SBE2 alone results in the intermediate phenotype discussed below. An additional moderate decrease in SBE1 does not alter this phenotype, but strong reductions in SBE1 as well as SBE2 result in an extreme phenotype. This is illustrated by comparison of lines 212‐36 and 227‐25 (intermediate phenotypes) and 237/4‐44 (extreme phenotype). The reduction in SBE2 protein is actually greater in 212‐36 and 227‐25 than in 237/4‐44, but whereas 212‐36 and 227‐25 have only moderate losses of SBE1 protein, 237/4‐44 has a very strong reduction in SBE1 protein.

Common features of extreme phenotypes seen in several studies include cracking across the hilum of the granule, depletion of the short‐chain fraction and large increases in the long chain fraction of amylopectin, and a decrease in branching frequency of 50% or more. These phenomena are consistent with reduced branching of nascent amylopectin at the granule surface, resulting in a preponderance of long chains and—in extreme cases—reduced amylopectin synthesis, presumably caused by the reduced level of free nonreducing ends of chains at the granule surface as substrates for starch synthases. Previous reports of very large increases in amylose content (up to 80% or more compared with wild‐type levels of 20%–30%) in severe *sbe* mutants may be due to the detection of long amylopectin chains by iodine‐based amylose assays and/or a low ratio of amylopectin to amylose in the starch because of reduced amylopectin synthesis.

Our most extreme mutant had a less severe phenotype than the most extreme lines reported previously by Schwall *et al*. (2000) and Hofvander *et al*. (2004). Their mutants had strong reductions in starch granule size, an almost complete loss of material from the amylopectin region of SEC chromatograms and severely reduced tuber starch yields. It seems likely that our most extreme line retained more functional SBE than their lines. Very small but readily detectable amounts of SBE1 and SBE2 protein were present in tubers of the extreme line 237/4‐44.

### Reduction in SBE2 produces a novel starch phenotype

We saw distinctive starch phenotypes in one line mutated only in SBE2 and two lines mutated in both SBE isoforms but retaining substantial SBE1 protein (lines 230‐51, 212‐36 and 227‐25). Starch granules from these intermediate lines did not have the cracks around the hilum seen in the extreme lines, but a high proportion appeared to have arisen from multiple hila. Polarized light microscopy revealed multiple Maltese crosses within granules with relatively normal shapes, and some granules had irregular nodular shapes apparently arising from the fusion of multiple small granules. Strikingly, individual tuber cells also contained huge numbers of tiny granules. These were not present in tubers of wild‐type plants or extreme mutant lines. The clustered distribution of the tiny granules in isolated cells suggests that they were present in only one or a few amyloplasts per cell.

Despite the obvious starch granule phenotype, the structure and degree of branching (measured by ^1^H NMR) of amylopectin in tuber starch were not strongly affected in the intermediate lines. Based on these observations, we propose that major loss of SBE2 has relatively minor impacts on amylopectin synthesis at the surface of growing granules, but strongly affects the process of granule initiation. The presence of huge numbers of tiny granules, granules with multiple hila and nodular granules points to a loss of control over the granule initiation process. Rather than initiation of a single granule per amyloplast as in wild‐type tubers (Ohad *et al*., 1971; Wetzstein and Sterling, 1978), at least some amyloplasts initiate huge numbers of granules. These may remain as independent structures, or fuse to form nodular granules, or fuse and then grow as a single granule with multiple hila. Recent research in Arabidopsis shows granule initiation to be a function of a multiprotein complex that contains starch synthase 4, an isoform of starch synthase required for initiation of wild‐type numbers of granules per chloroplast. It is proposed that clusters of these complexes produce glucan chains that associate to form granule initials that are substrates for chain‐elongating and branching enzymes, leading to granule growth (Seung and Smith, 2018; Seung *et al*., 2017, 2018). Individual mis‐expression of several enzymes involved in granule initiation and elaboration in Arabidopsis leaves results in initiation of excessive numbers of granules (Delatte *et al*., 2005; Feike *et al*., 2016; Seung *et al*., 2017). Intriguingly, strong reduction in potato tubers of the debranching enzyme isoamylase produced a phenotype similar to that produced by reduction in the branching enzyme SBE2. Individual tuber cells in isoamylase‐deficient plants accumulated both nodular granules and very large numbers of tiny granules (Bustos *et al*., 2004). We speculate that inappropriate levels of branching of glucan chains emerging from initiation complexes prevent the operation of mechanisms that limit numbers of initiations, allowing large, variable numbers of initiations to occur.

### Cas9‐mediated mutagenesis holds promise for development of commercially viable potato varieties with elevated levels of resistant starch

Our results extend and support recent demonstrations that Cas9‐mediated mutagenesis in potato can produce a wide range of mutations with diverse phenotypic consequences in potentially transgene‐free plants. Previous research on potatoes with reduced SBE activity has necessitated introduction of transgenes and has focussed on extreme starch phenotypes. Although these starches are resistant to digestion (Zhao *et al*., 2018), the transgenic plants producing them have altered field performance, tuber composition, dry matter content and processability. These genotypes are unlikely to be commercially acceptable. We suggest that screening of plants with Cas9‐induced targeted mutations, carrying a range of different *SBE* alleles, can potentially uncover new variation in starch properties. Unlike techniques used previously to reduce SBE activity in potatoes, Cas9‐mediated mutagenesis can be directed to regions of *SBE* genes that encode domains involved in—for example—catalysis, glucan binding, interaction with other starch‐synthesizing proteins or substrate affinity (Chaen *et al*., 2012; Li and Gilbert, 2016), thus permitting a much greater range of potential changes in SBE function. It is entirely feasible that mutants will be found with nutritionally relevant increases in resistant starch and no other deleterious changes. Recent developments in CRISPR‐derived technology for potatoes promise to improve the efficiency of generation of transgene‐free mutants (Andersson *et al*., 2017, 2018; Kusano *et al*., 2018), thus allowing large‐scale screens for nutritionally desirable phenotypes.

## Experimental procedures

### Identification and cloning of *SBE1* and *SBE2* from *Solanum tuberosum* cv Desiree

The translated coding sequence of *SBE1* (GenBank ID: Y08786; Khoshnoodi *et al*., 1996) was used to query the transcriptome of the reference potato genome *S. tuberosum* Group Phureja clone DM1‐3 516R44 (DM) (The Potato Genome Sequencing Consortium, 2011) using tBLASTn at the Sol Genomics database (https://solgenomics.net). A candidate transcript (id PGSC0003DMC400017627) mapped to scaffold PGSC0003DMS000001367. The translated coding sequence of *SBE2* (GenBank ID: CAB40748; Jobling *et al*., 1999) was used to query the transcriptome, resulting in a candidate transcript that mapped to scaffold PGSC0003DMS000000876. Gene structure was inferred using GeneWise (https://www.ebi.ac.uk/Tools/psa/genewise/). A third candidate SBE (SBE3; Sotub07g029010.1.1) was identified by tBLASTn analysis of the transcriptome and BLASTp analysis of the predicted proteins.

The reference genome was used to design primers to amplify and clone cDNAs encoding *SBE1* and *SBE2* from *S. tuberosum* cv. Desiree (Table S2). RNA from leaves (RNeasy Plant Mini Kit; Qiagen, http://www.quiagen.com) was used for first‐strand cDNA synthesis (SuperScript II RT; Invitrogen, http://www.invitrogen.com) from which *SBE1* and *SBE2* were amplified (40 cycles at 95 °C for 30 s, 55 °C for 30 s and 72 °C for 3 min) using Phusion polymerase (NEB, http://www.neb.com). PCR amplicons were cloned into PCR8/GW/TOPO TA (Invitrogen) according to the manufacturer's instructions. Eight clones of each gene were sequenced. Multiple sequence alignment (Clustal Omega, http://www.clustal.org/omega) identified single nucleotide polymorphisms consistent with the presence of two homoeologues of each gene (Figure S2), as expected for a tetraploid genome.

### Selection of targets and construction of plasmids for Cas9‐induced targeted mutagenesis

Target sites adjacent to NGG protospacer‐adjacent motifs (PAMs) were identified for Cas9‐induced mutagenesis of *SBE1* and *SBE2*. Isoform specificity was ensured by selection of targets that differed in at least five bases (Figure S1, Table S3).

Plasmid vectors for Cas9‐mediated targeted mutagenesis were constructed according to Lawrenson *et al*. (2015) using the Plant Modular Cloning Golden Gate toolkit, a gift from Sylvestre Marillonnet (Addgene #1000000044; Engler *et al*., 2014). Briefly, targets were introduced into single guide RNA (sgRNA) scaffolds by PCR (for primers, see Table S2) and assembled with a U6 small RNA promoter from either Arabidopsis (plCSL90002; Addgene #68261) or potato (Wang *et al*., 2015) into a Level 1 acceptor plasmid (plCH47751, plCH47761, plCH47772 or plCH47781). The guide sequences in the sgRNAs follow the consensus G(N)_19–20_ where the first G, for initiation of transcription from the U6 promoter, may or may not pair up with the target DNA sequence (Belhaj *et al*., 2013). The potato U6 promoter was synthesized (Integrated DNA Technologies, http://www.idtdna.com), mutating the instance of the *BbsI* recognition site ‘gaagac’ to ‘gtagac’, and cloned into pUAP1 (Addgene # 63674) to create a new Level 0 part. The Level 1 assemblies containing sgRNA expression cassettes were assembled into the Level M Position 1 acceptor (pAGM8031; Addgene #48073) together with a kanamycin resistance cassette (pICSL11055; Addgene #68252) and a Cas9 expression cassette either with (pICSL11023; Addgene #117542) or without a YFP C‐terminal tag (plCSL11021; Addgene #49771, a kind gift from Sophien Kamoun; Sainsbury Laboratory, Norwich, UK). Twelve plasmids with different combinations of U6 promoters and sgRNAs were constructed (Figure S3).

### *Agrobacterium*‐mediated transformation

Plasmid constructs were transformed into *Agrobacterium tumefaciens* AGL1 by electroporation, and single colonies were propagated at 28 °C on solid or liquid LB selective media (rifampicin 50 μg/mL; spectinomycin 50 μg/mL). Culture preparation, transformation and regeneration of potato plants were performed according to Chetty *et al*. (2015). Pathogen‐free nuclear stocks of *S. tuberosum* cv. Desiree used for transformation were from Science and Advice for Scottish Agriculture (SASA, Edinburgh, UK). When regenerated shoots were ~10 cm, tissue was sampled for genotyping. Plants were transferred to potting mix after clonal propagation from axillary buds.

### Protoplast transformation

Polyethylene glycol (PEG)‐mediated protoplast transformation and plant regeneration were performed according to Nicolia *et al*. (2015) with minor modifications. Briefly, protoplasts were prepared from leaves of 4‐week‐old plantlets grown in MS30 with 0.8% agar and purified by filtration and centrifugation onto a 0.43 m cushion of sucrose. Protoplast viability was checked by staining a sample with fluorescein diacetate and propidium iodide, and protoplast concentrations were adjusted to 1.5 × 10^6^ protoplasts/mL. Samples of 200 μL of protoplast suspension were incubated for 5 min with 20 μL of plasmid DNA (1 μg/μL) and 220 μL of PEG solution. As a control, 20 μL dH_2_O was used in place of plasmid DNA. Parallel transformations were performed using constructs encoding Cas9 with or without a C‐terminal YFP tag (Figure S3).

After transformation, protoplasts were resuspended in nutrient‐rich liquid medium E (Nicolia *et al*., 2015) to a final concentration of 10^5^ protoplasts/mL. After 3 days, samples transfected with plasmids encoding a Cas9:YFP fusion protein were examined by fluorescence microscopy to calculate transformation efficiency (Figure S6a) and gDNA was extracted and assessed for mutations in the target genes by PCR (described below, Figure S6b). Where transformation efficiency was ≥20%, protoplasts were allowed to proliferate further then transferred to medium F and G as described by Nicolia *et al*. (2015). Calli of 1–2 mm were transferred from medium G onto callus induction medium (MS20 containing 0.8% (w/v) agar, 2.5 mg/L zeatin, 0.2 mg/L NAA, 0.02 mg/L GA_3_, 80 mg/L timentin and 125 mg/L cefotaxime). After 2 weeks, strongly growing calli were transferred to shoot induction medium (as above but with 2.0 mg/L zeatin, 0.02 mg/L NAA) and subcultured every 2 weeks. Samples were taken for genotyping. Shoots from calli with putative mutations in *SBE* genes were excised and grown on solid rooting medium (MS30 containing 0.8% (w/v) agar) without antibiotics before transfer to potting mix.

### Characterization of induced mutations

Genomic DNA (gDNA) was purified from leaves using the DNeasy 96 plant kit (Qiagen) or from calli as follows. Samples of 20 mg were crushed in 250 μL 200 mm Tris–HCl, pH 7.5, 250 mm NaCl, 25 mm EDTA, 0.5% (w/v) SDS; incubated at 65 °C for 15 min; mixed with 250 μL phenol and the upper aqueous phase collected following centrifugation (18 000 ***g***, 10 min); and extracted with an equal volume of chloroform. After centrifugation DNA was precipitated with 100 μL isopropanol, collected by centrifugation, washed in 1 mL 70% (v/v) ethanol and resuspended in 50 μL dH_2_O.

The presence/absence of the Cas9 gene was checked by PCR with Cas9‐specific primers (Table S2) on plants in tissue culture or in the glasshouse, following *Agrobacterium*‐mediated and protoplast transformation, respectively. Primers specific for *SBE1* or *SBE2* were used to detect Cas9‐induced mutations at the target. Amplicons were analysed by Sanger sequencing to determine the exact genotype. Putative mutants were propagated in a glasshouse, and genotyping was repeated on mature plants. Plants regenerated from protoplasts which were mock‐transformed with water in place of DNA were used as negative controls.

### Growth conditions and plant harvesting

Regenerated plants were grown in soil in a glasshouse with 22 °C day, 16 °C night and natural daylight. Protoplast‐derived plants were grown to maturity in 10‐L pots; plants derived from *Agrobacterium*‐mediated transformation were grown in 50‐L bags. Tubers were harvested shortly before or during plant senescence. Samples for starch assays and immunoblotting were taken from freshly harvested tubers using a corkborer, immediately frozen in liquid nitrogen and stored at −70 °C.

### Starch extraction, assay and structural analysis

Starch was prepared from tubers (30–100 g) according to Edwards *et al*. (1995). Starch granule size and volume distributions were measured with a Multisizer 4e Coulter counter with a 200 μm aperture (Beckman Coulter Life Sciences, http://www.beckman.com), according to the manufacturer's instructions.

Chain‐length distributions were analysed by HPLC‐SEC following solubilization and enzymatic debranching of purified starch, according to Perez‐Moral *et al*. (2018). ^1^H‐NMR analysis of branch‐point frequency was performed according to Tizzotti *et al*. (2011) and Schmitz *et al*. (2009). Details of ^1^H NMR and HPLC‐SEC are in Appendix S1.

Measurement of tuber starch content was carried out according to Smith and Zeeman (2006) except that samples of frozen tuber were homogenized in 1 m perchloric acid. After centrifugation, the pellet was washed and then heated to solubilize starch. Starch was hydrolysed with α‐amyloglucosidase and α‐amylase, and glucose was assayed enzymatically.

### Immunoblotting

Immunoblotting was carried out as described by Jobling *et al*. (1999). Isoform‐specific antisera were a kind gift from Unilever Research (Colworth House, Bedford, UK). Note that SBE A and SBE B described by Jobling *et al*. (1999) are SBE2 and SBE1, respectively.

### Light microscopy

For cell separation (Mandalari *et al*., 2018; McCartney and Knox, 2002; Parker and Guerra, 2008), tuber slices of ≤0.5 mm thickness were incubated in 50 mm 1,2‐cyclohexylenedinitrilotetraacetic acid (CDTA), 5 mm Na_2_S_2_O_5_ (pH 7.0) for 3 days. Cells were separated on a slide by gentle pressure with a coverslip. Starch granules were observed with bright field and polarized light microscopy, either unstained or after staining with appropriately diluted (between twofold and 10‐fold) Lugol's iodine (2%).

## Conflict of interest

The authors declare no conflict of interests.

## Author contribution

AT, NJP and AMS devised the project; AT and NJP designed the constructs. MAS and AT with advice from WH generated the mutated plants. AT characterized mutations and starch granules; EH measured starch and granule size distributions and performed immunoblots. FJW, KRC, SH and JA‐J performed HPLC‐SEC and NMR analyses. The manuscript was written by AMS, AT and NJP, and corrected and improved by all the authors.

## Supporting information


**Figure S1** Schematic presentations of the *SBE1* and *SBE2* genes showing exons and sgRNA targets for Cas9‐mediated mutagenesis.
**Figure S2** Alignment of coding sequences of two copies of *SBE1* and *SBE2*.
**Figure S3** Design of constructs for RNA‐guided Cas9‐mediated mutagenesis of *SBE* genes.
**Figure S4** PCR analysis of *SBE1* in a selection of lines generated by a, *Agrobacterium*‐mediated transformation; B, PEG‐mediated protoplast transformation.
**Figure S5** PCR analysis of *SBE2* in selection of lines generated by (a) *Agrobacterium*‐mediated transformation and (b) PEG‐mediated protoplast transformation.
**Figure S6** Examples of confirmation of Cas9 expression and mutation of *SBE* genes in protoplasts 3 days after PEG‐mediated protoplast transformation.
**Figure S7** Detection of Cas9 transgene in protoplast‐derived plants.
**Figure S8** Sequence analysis of *SBE1* from line 237/4‐44.
**Figure S9** Sequence analysis of *SBE2* from line 237/4‐44.
**Figure S10** Sequence analysis of *SBE2* from line 230‐51a and b.
**Figure S11** Sequence analysis of *SBE1* from line 227‐25.
**Figure S12** Sequence analysis of *SBE1* from line 212‐36.
**Figure S13** Sequence analysis of *SBE2* from line 212‐36.
**Figure S14** Sequence analysis of *SBE2* from line 227‐25.
**Figure S15** Images of tuber starch granules from mutant and wild‐type lines.
**Figure S16** Starch contents of mature tubers.
**Table S1** Numbers of plants with mutations at the intended targets.
**Table S2** Primers used in this study.
**Table S3** sgRNA recognition sequences in *SBE1* and *SBE2*.
**Appendix S1** Supplemental Experimental Procedures: ^1^H NMR and HPLC‐SEC.
