# Supplementary material for: Cas9‐mediated mutagenesis of potato starch‐branching enzymes generates a range of tuber starch phenotypes
Source: Plant Biotechnol J. 2019 May 14;17(12):2259–71. doi: 10.1111/pbi.13137 (PMC6835119; doi:10.1111/pbi.13137)
Supplement: Supplementary file 1 — Figure S1 Schematic presentations of the SBE1 and SBE2 genes showing exons and sgRNA targets for Cas9‐mediated mutagenesis. Figure S2 Alignment of coding sequences of two copies of SBE1 and SBE2. Figure S3 Design of constructs for RNA‐guided Cas9‐mediated mutagenesis of SBE genes. Figure S4 PCR analysis of SBE1 in a selection of lines generated by a, Agrobacterium‐mediated transformation; B, PEG‐mediated protoplast transformation. Figure S5 PCR analysis of SBE2 in selection of lines generated by (a) Agrobacterium‐mediated transformation and (b) PEG‐mediated protoplast transformation. Figure S6 Examples of confirmation of Cas9 expression and mutation of SBE genes in protoplasts 3 days after PEG‐mediated protoplast transformation. Figure S7 Detection of Cas9 transgene in protoplast‐derived plants. Figure S8 Sequence analysis of SBE1 from line 237/4‐44. Figure S9 Sequence analysis of SBE2 from line 237/4‐44. Figure S10 Sequence analysis of SBE2 from line 230‐51a and b. Figure S11 Sequence analysis of SBE1 from line 227‐25. Figure S12 Sequence analysis of SBE1 from line 212‐36. Figure S13 Sequence analysis of SBE2 from line 212‐36. Figure S14 Sequence analysis of SBE2 from line 227‐25. Figure S15 Images of tuber starch granules from mutant and wild‐type lines. Figure S16 Starch contents of mature tubers. Table S1 Numbers of plants with mutations at the intended targets. Table S2 Primers used in this study. Table S3 sgRNA recognition sequences in SBE1 and SBE2. Appendix S1 Supplemental Experimental Procedures: 1H NMR and HPLC‐SEC. [file PBI-17-2259-s001.docx]

**Cas9-mediated mutagenesis of potato starch branching enzymes generates a range of tuber starch phenotypes**

Aytug Tuncel, Kendall L. Corbin, Jennifer Ahn-Jarvis, Erica Hawkins, Mark Smedley, Wendy Harwood, Frederick J. Warren, Nicola J. Patron^*^, Alison M. Smith^*^

**Supporting Information**

**Table S1.** Numbers of plants with mutations at the intended targets.

**Table S2.** Primers used in this study.

**Table S3.** sgRNA recognition sequences in *SBE1* and *SBE2*.

**Figure S1.** Schematic presentations of the *SBE1* and *SBE2* genes showing exons and sgRNA targets for Cas9-mediated mutagenesis.

**Figure S2.** Alignment of coding sequences of two copies (possible homeoalleles) of **a,** *SBE1* and **b**, *SBE2*.

**Figure S3.** Design of constructs for RNA-guided Cas9-mediated mutagenesis of *SBE* genes.

**Figure S4.** PCR analysis of *SBE1* in a selection of lines generated by **a**, *Agrobacterium*-mediated transformation and **b**, PEG-mediated protoplast transformation.

**Figure S5.** PCR analysis of *SBE2* in selection of lines generated by **a**, *Agrobacterium*-mediated transformation and **b**, PEG-mediated protoplast transformation.

**Figure S6.** Examples of confirmation of Cas9 expression and mutation of *SBE* genes in protoplasts three days after PEG-mediated protoplast transformation.

**Figure S7.** Detection of Cas9 transgene in protoplast-derived plants.

**Figure S8.** Sequence analysis of *SBE1* from line 237/4-44.

**Figure S9.** Sequence analysis of *SBE2* from line 237/4-44.

**Figure S10.** Sequence analysis of *SBE2* from line 230-51a and b.

**Figure S11.** Sequence analysis of *SBE1* from line 227-25.

**Figure S12.** Sequence analysis of *SBE1* from line 212-36.

**Figure S13.** Sequence analysis of *SBE2* from line 212-36.

**Figure S14.** Sequence analysis of *SBE2* from line 227-25.

**Figure S15:** Images of tuber starch granules from mutant and wild-type lines.

**Figure S16.** Starch contents of mature tubers.

**Supporting Experimental Procedures**: ^1^H NMR and HPLC-SEC

**Table S1**. Numbers of plants with mutations at the intended targets.

The upper table shows plants recovered from *Agrobacterium*-mediated transformation of stem explants. The lower table shows mutated plants recovered from PEG-mediated transformation of leaf-derived protoplasts.

| Construct | Target | Number of plants recovered | Plants with mutations in *SBE1* only | Plants with mutations in *SBE2* only | Plants with mutations in *SBE1* and *SBE2* | Number of plants without Cas9 transgene |  |
| --- | --- | --- | --- | --- | --- | --- | --- |
| pAT212 | *SBE1* + *SBE2* | 74 | - | 2 | 2 | 0 |  |
| pAT227 | *SBE1* + SBE2 | 200 | 2 | 6 | 5 | 0 |  |
| pAT213 | *SBE1* | 67 | 6 | NA | NA | 0 |  |
| pAT216 | *SBE1* | 162 | 6 | NA | NA | 0 |  |
| pAT214 | *SBE2* | 40 | NA | 10 | NA | 0 |  |
| pAT230 | *SBE2* | 224 | NA | 6 | NA | 0 |  |
|  |  |  |  |  |  |  |  |
| Construct | Target | Number of calli recovered | Calli with mutations in *SBE1* only | Calli with mutations in *SBE2* only | Calli with mutations in *SBE1* and *SBE2* | Number of plants | Number of plants without Cas9 transgene |
| pAT237 | *SBE1* + *SBE2* | 362 | - | - | 7 | 19 | 11 |
| pAT239 | *SBE1* | 141 | 2 | NA | NA | 3 | 3 |
| pAT241 | S*BE2* | 162 | NA | 1 | NA | 3 | 3 |

**Table S2.** Primers used in this study.

Target sites from *SBE1* or *SBE2* are in upper case. *BsaI* recognition sites are underlined.

| **Name** | **Sequence (5’ – 3’)** | **Purpose** |
| --- | --- | --- |
| oAT214 | tgtggtctcatcgGTTGCATAGTCTATCGTGAA  gttttagagctagaaatagcaag | Produce sgRNA-1 scaffold to be fused with S. tuberosum U6 promoter |
| oAT212 | tgtggtctcatcgGAAGTTATTGGCGATTTCAA  gttttagagctagaaatagcaag | Produce sgRNA-2 scaffold to be fused with S. tuberosum U6 promoter |
| oAT237 | tgtggtctcatcgGTTCAACTATGCCAATTGGG  gttttagagctagaaatagcaag | Produce sgRNA-3 scaffold to be fused with S. tuberosum U6 promoter |
| oAT238 | tgtggtctcatcggCAATGGCAATCCCAGATAAG  gttttagagctagaaatagcaag | Produce sgRNA-4 scaffold to be fused with S. tuberosum U6 promoter |
| oAT216 | tgtggtctcatcgGCAGAATGAATTTGGTGTCT  gttttagagctagaaatagcaag | Produce sgRNA-5 scaffold to be fused with S. tuberosum U6 promoter |
| oAT217 | tgtggtctcatcggTTAAGGATTCCATTCCTGCT  gttttagagctagaaatagcaag | Produce sgRNA-6 scaffold to be fused with S. tuberosum U6 promoter |
| oAT260 | tgtggtctcatcgGCATCAAATAATACTTTAGA  gttttagagctagaaatagcaag | Produce sgRNA-7 scaffold to be fused with S. tuberosum U6 promoter |
| oAT241 | tgtggtctcatcgGGGATTCACTGGGAACTACG  gttttagagctagaaatagcaag | Produce sgRNA-9 scaffold to be fused with S. tuberosum U6 promoter |
| oAT240 | tgtggtctcatcggTTCCCAGATGCAATTACCAT  gttttagagctagaaatagcaag | Produce sgRNA-10 scaffold to be fused with S. tuberosum U6 promoter |
| oAT2 | tgtggtctcaATTGTTGCATAGTCTATCGTGAA  gttttagagctagaaatagcaag | Produce sgRNA-1 scaffold to be fused with A. thaliana U6-26 promoter |
| oAT6 | tgtggtctcaATTGAAGTTATTGGCGATTTCAA  gttttagagctagaaatagcaag | Produce sgRNA-2 scaffold to be fused with A. thaliana U6-26 promoter |
| oAT16 | tgtggtctcaattgATCTTCTCTCAAATGCGAGA  gttttagagctagaaatagcaag | Produce sgRNA-8 scaffold to be fused with A. thaliana U6-26 promoter |
| oAT17 | tgtggtctcaATTGGGATTCACTGGGAACTACG  gttttagagctagaaatagcaag | Produce sgRNA-9 scaffold to be fused with A. thaliana U6-26 promoter |
| oAT1 | tgtggtctcaagcgtaatgccaactttgtac | Reverse primer used to produce all the sgRNA scaffolds |
| oAT138-F | ccttctgttgcttcacaatttcc | amplification of *SBE1* to identify mutations at targets |
| oAT123-R | cccagtagacaccatcatatggtgc |  |
| oAT36-R | caaactcgatgttacttccttc |  |
| oAT148-R | catgaatcgaaatgcctgtgatg |  |
| oAT184-F | agatggtggttggatgagttc | amplification of *SBE2* to identify mutations at targets |
| oAT55-F | gcaccaagcagccgttttgg |  |
| oAT139-F | ggaagtctttgcaagaaaatgtgg |  |
| oAT134-F | gctcgtggttatcattggatgtgg |  |
| oAT244-R | ctccagcttgatgatcctattatc |  |
| oAT168-F | caaggacttcctggacaatgaggag | screening for presence of *cas9* transgene |
| oAT169-R | tcatcgtacttggtgttcatgcgtg |  |
| oAT151-F | gaagcaaagtaccataatttaatc | amplification of *SBE1* from cDNA (5' UTR) |
| oAT154-R | ggaccgagatcaccaacgtagaagg | amplification of *SBE1* from cDNA (3' UTR) |
| oAT155-F | taaaaaccctaaggagagaagaag | amplification of *SBE2* from cDNA (5' UTR) |
| oAT158-R | ccagatacgtcaagaagctctatg | amplification of *SBE2* from cDNA (3' UTR) |
| oAT159-F | cagggaagatggttgcatagtc | Sequencing of *SBE1* |
| oAT160-F | gatggccataatggaacattc |  |
| oAT162-F | ctcgcagatagcgaacacttgag |  |
| oAT170-F | caactgttattgccgaagatg |  |
| oAT172-F | gatttcaatggatggaacgg |  |
| oAT173-F | aggacctaaagtatctgatag |  |
| oAT175-F | ctgagggaggaattggttttg |  |
| oAT177-F | gttctcattcctcgcatcagg |  |
| oAT164-F | cactacaactacaagaaggtgg | Sequencing of *SBE2* |
| oAT165-F | ccattcctgcttggatcaactac |  |
| oAT166-F | ctatagcattctggctgatggac |  |
| oAT181-F | ccacctggacttggtcagaag |  |
| oAT182-F | cttccaacacccacggccaaag |  |
| oAT183-R | ctcattccaatatgagattc |  |
| oAT184-F | agatggtggttggatgagttc |  |
| oAT185-F | cattaatagatcgtgggatagc |  |

**Table S3.** sgRNA recognition sequences in *SBE1* and *SBE2*.

Protospacer adjacent motifs (PAMs) are underlined.

| **Target gene** | **Name of sgRNA** | **Target sequence (5' – 3')** |
| --- | --- | --- |
| ***SBE1*** | sgRNA-1 | gttgcatagtctatcgtgaatgg |
|  | sgRNA-2 | gaagttattggcgatttcaatgg |
|  | sgRNA-3 | gttcaactatgccaattgggagg |
|  | sgRNA-4 | caatggcaatcccagataagtgg |
| ***SBE2*** | sgRNA-5 | gcagaatgaatttggtgtctggg |
|  | sgRNA-6 | ttaaggattccattcctgcttgg |
|  | sgRNA-7 | gcatcaaataatactttagatgg |
|  | sgRNA-8 | atcttctctcaaatgcgagatgg |
|  | sgRNA-9 | gggattcactgggaactacgagg |
|  | sgRNA-10 | ttcccagatgcaattaccattgg |

**Figure S1.** Schematic presentations of the *SBE1* and *SBE2* genes showing exons and sgRNA targets for Cas9-mediated mutagenesis. Exons are indicated by numbered arrows. Nucleotides that differ between the *SBE* isoforms are shaded. Protospacer adjacent motifs (PAMs) are underlined.


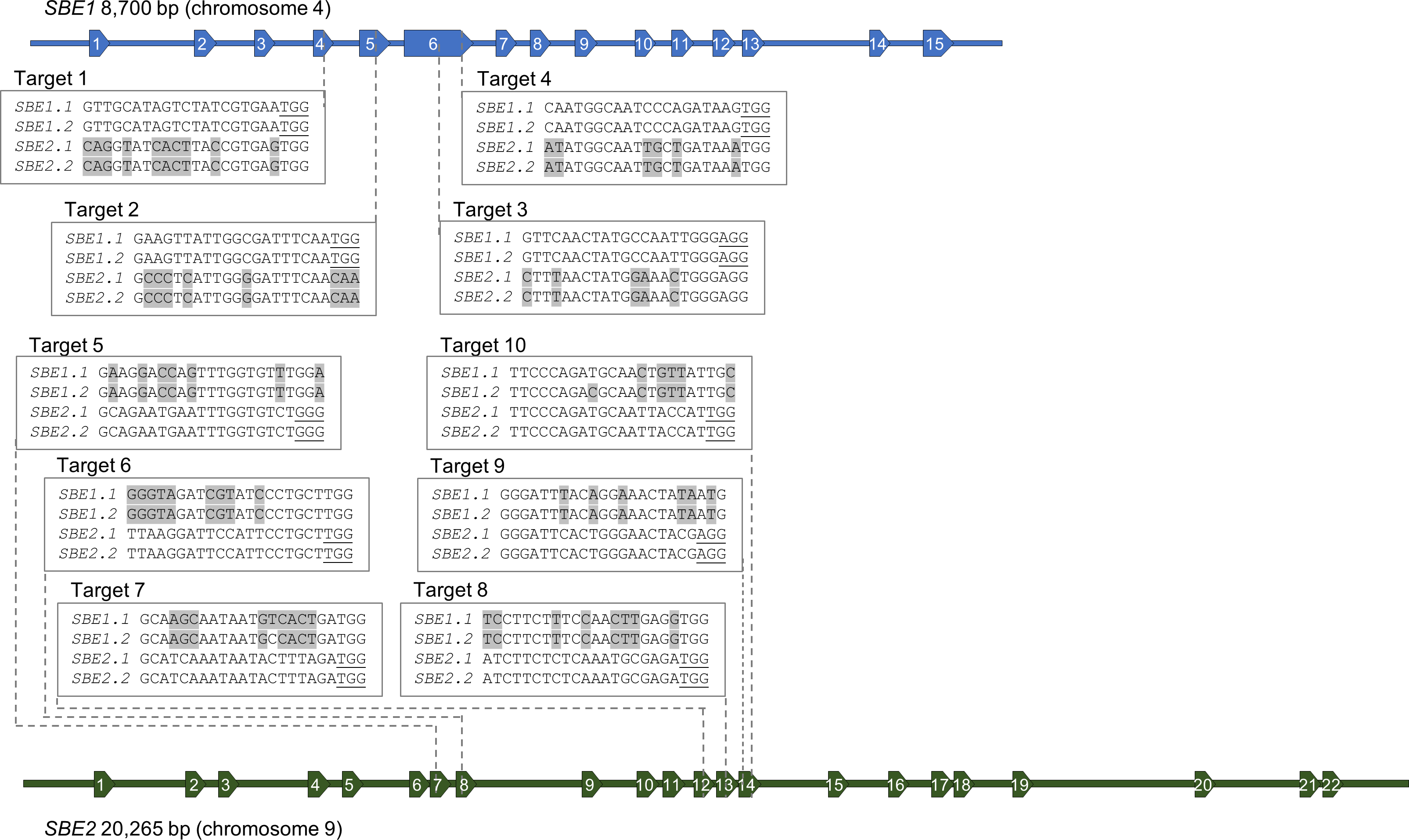


**Figure S2.** Alignment of coding sequences of two copies (possible homoeoalleles) of **a,** *SBE1* and **b**, *SBE2*.

Differences are shown by coloured shading.

**a**

SBE1.2 ATGGAAATTAATTTCAATGTTTTGTCAAAACCCATTCGAGGATCTTTTCCATCTTCCTCA 60

SBE1.1 ATGGAAATTAATTTCAAAGTTTTATCAAAACCCATTCGAGGATCTTTTCCATCTTTCTCA 60

SBE1.2 CCTAAAGTTTCTTCAGGGGCTTCTAGAAATAAGATATGTTTTCCTTCTCAACATAGTACT 120

SBE1.1 CCTAAAGTTTCTTCAGGGGCTTCTAGAAATAAGATATGTTTTCCTTCTCAACATAGTACT 120

SBE1.2 GGACTGAAGTTTGGATCTCAGGAACGGTCTTGGGATATTTCTTCCACCCCAAAATCAAGA 180

SBE1.1 GGACTGAAGTTTGGATCTCAGGAACGGTCTTGGGATATTTCTTCCACCCCAAAATCAAGA 180

SBE1.2 GTTAGAAAAGATGAAAGGATGAAGCACAGTTCAGCTATTTCCGCTGTTTTGACCGATGAC 240

SBE1.1 GTTAGAAAAGATGAAAGGATGAAGCACAGTTCAGCTATTTCCGCTGTTTTGACCGATGAC 240

SBE1.2 AATTCGACAATGGCACCCCTAGAGGAAGATGTCAAGACTGAAAATATTGACCTCCTAAAT 300

SBE1.1 AATTCGACAATGGCACCCCTAGAGGAAGATGTCAAGACTGAAAATATTGGCCTCCTAAAT 300

SBE1.2 TTGGATCCAGCTTTGGAACCTTATCTAGATCACTTCAGATACAGAATGAAGAGATATGTG 360

SBE1.1 TTGGATCCAACTTTGGAACCTTATCTAGATCACTTCAGACACAGAATGAAGAGATATGTG 360

SBE1.2 GATCAGAAAATGCTCATTGAAAAATATGAGGGACCCCTTGAGGAATTTGCTCAAGGTTAT 420

SBE1.1 GATCAGAAAATGCTCATTGAAAAATATGAGGGACCCCTTGAGGAATTTGCTCAAGGTTAT 420

SBE1.2 TTAAAATTTGGATTCAACAGGGAAGATGGTTGCATAGTCTATCGTGAATGGGCTCCTGCT 480

SBE1.1 TTAAAATTTGGATTCAACAGGGAAGATGGTTGCATAGTCTATCGTGAATGGGCTCCTGCT 480

SBE1.2 GCTCAGGAAGCAGAAGTTATTGGCGATTTCAATGGATGGAACGGTTCCAACCACATGATG 540

SBE1.1 GCTCAGGAAGCAGAAGTTATTGGCGATTTCAATGGATGGAACGGTTCTAACCACATGATG 540

SBE1.2 GAGAGGGACCAGTTTGGTGTTTGGAGTATTAGAATTCCTGATGTTGACAGTAAGCCAGTC 600

SBE1.1 GAGAAGGACCAGTTTGGTGTTTGGAGTATTAGAATTCCTGATGTTGACAGTAAGCCAGTC 600

SBE1.2 ATTCCACACAACTCCAGAGTTAAGTTCCGTTTCAAACATGGTAATGGAGTGTGGGTAGAT 660

SBE1.1 ATTCCACACAACTCCAGAGTTAAGTTTCGTTTCAAACATGGTAATGGAGTGTGGGTAGAT 660

SBE1.2 CGTATCCCTGCTTGGATAAAGTATGCCACTGAAGACTCCACAAAGTTTGCAGCACCATAT 720

SBE1.1 CGTATCCCTGCTTGGATAAAGTATGCCACTGCAGACGCCACAAAGTTTGCAGCACCATAT 720

SBE1.2 GATGGTGTCTACTGGGACCCACCACCTTCAGAAAGGTACCACTTCAAATACCCTCGCCCT 780

SBE1.1 GATGGTGTCTACTGGGACCCACCACCTTCAGAAAGGTACCACTTCAAATACCCTCGCCCT 780

SBE1.2 CCCAAACCCCGAGCCCCACGAATCTACGAAGCACATGTCGGCATGAGCAGCTCTGAGCCA 840

SBE1.1 CCCAAACCCCGAGCCCCACGAATCTATGAAGCACATGTCGGCATGAGCAGCTCTGAGCCA 840

SBE1.2 CGTGTAAATTCATATCGTGAGTTTGCAGATGATGTTTTACCTCGGATTAAGGCAAATAAC 900

SBE1.1 CGTGTAAATTCGTATCGTGAGTTTGCAGATGATGTTTTACCTCGGATTAAGGCAAATAAC 900

SBE1.2 TATAATACTGTCCAGTTGATGGCCATAATGGAACATTCTTACTATGGATCATTTGGATAT 960

SBE1.1 TATAATACTGTCCAGTTGATGGCCATAATGGAACATTCTTACTATGGATCATTTGGATAT 960

SBE1.2 CATGTTACAAACTTTTTTGCTGTGAGCAGTAGATATGGAAACCCGGAGGACCTAAAGTAT 1020

SBE1.1 CATGTTACAAACTTTTTTGCTGTGAGCAGTAGATGTGGAAACCCGGAGGACCTAAAGTAT 1020

SBE1.2 CTGATAGATAAAGCACATAGCTTGGGTTTACAGGTTCTGGTGGATGTAGTTCACAGTCAT 1080

SBE1.1 CTGATAGATAAAGCACATAGCTTGGGTTTACAGGTTCTGGTGGATGTAGTTCACAGTCAT 1080

SBE1.2 GCAAGCAATAATGCCACTGATGGCCTCAATGGCTTTGATATTGGCCAAGGTTCTCAAGAA 1140

SBE1.1 GCAAGCAATAATGTCACTGATGGCCTCAATGGCTTTGATATTGGCCAAGGTTCTCAAGAA 1140

SBE1.2 TCCTACTTTCATGCTGGAGAGCAAGGGTACCATAAGTTGTGGGATAGCAGGCTGTTCAAC 1200

SBE1.1 TCCTACTTTCATGCTGGAGAGCGAGGGTACCATAAGTTGTGGGATAGCAGGCTGTTCAAC 1200

SBE1.2 TATGCCAATTGGGAGGTTCTTCGTTTCCTTCTTTCCAACTTGAGGTGGTGGCTAGAAGAG 1260

SBE1.1 TATGCCAATTGGGAGGTTCTTCGTTTCCTTCTTTCCAACTTGAGGTGGTGGCTAGAAGAG 1260

SBE1.2 TATAACTTTGACGGATTTCGATTTGATGGAATAACTTCTATGCTGTATGTTCATCATGGA 1320

SBE1.1 TATAACTTTGACGGATTTCGATTTGATGGAATAACTTCTATGCTGTATGTTCATCATGGA 1320

SBE1.2 ATCAATATGGGATTTACAGGAAACTATAATGAGTATTTCAGCGAGGCTACAGATGTTGAT 1380

SBE1.1 ATCAATATGGGATTTACAGGAAACTATAATGAGTATTTCAGCGAGGCTACAGATGTTGAT 1380

SBE1.2 GCTGTGGTCTATTTAATGTTGGCCAATAATCTGATTCACAAGATTTTCCCAGACGCAACT 1440

SBE1.1 GCTGTGGTCTATTTAATGTTGGCCAATAATCTGATTCACAAGATTTTCCCAGATGCAACT 1440

SBE1.2 GTTATTGCCGAAGATGTTTCTGGTATGCCGGGCCTTGGCCGGCCTGTTTCTGAGGGAGGA 1500

SBE1.1 GTTATTGCCGAAGATGTTTCTGGTATGCCGGGCCTTGGCCGGCCTGTTTCTGAGGGAGGA 1500

SBE1.2 ATTGGTTTTGATTACCGCCTGGCAATGGCAATCCCAGATAAGTGGATAGATTATTTAAAG 1560

SBE1.1 ATTGGTTTTGATTACCGCCTGGCAATGGCAATCCCAGATAAGTGGATAGATTATTTAAAG 1560

SBE1.2 AATAAAAATGATGAAGATTGGTCCATGAAGGAAGTAACATCGAGTTTGACAAATAGGAGA 1620

SBE1.1 AATAAGAATGATGAAGATTGGTCCATGAAGGAAGTAACATCGAGTTTGACAAATAGGAGA 1620

SBE1.2 TATACAGAGAAGTGTATAGCATATGCGGAGAGCCATGATCAGTCTATTGTCGGTGACAAG 1680

SBE1.1 TATACAGAGAAGTGTATAGCATATGCGGAGAGCCATGATCAGTCTATTGTTGGTGACAAG 1680

SBE1.2 ACCATTGCATTTCTCCTAATGGACAAAGAGATGTATTCTGGCATGTCTTGCTTGACAGAT 1740

SBE1.1 ACCATTGCATTTCTCCTAATGGACAAAGAGATGTATTCTGGCATGTCTTGCTTGACAGAT 1740

SBE1.2 GCTTCTCCTGTTATTGATCGAGGAATTGCGCTTCACAAGATGATCCATTTTTTCACAATG 1800

SBE1.1 GCTTCTCCTGTTGTTGATCGAGGAATTGCGCTTCACAAGATGATCCATTTTTTCACAATG 1800

SBE1.2 GCCTTGGGAGGAGAGGGGTACCTCAATTTCATGGGTAACGAGTTTGGCCATCCTGAGTGG 1860

SBE1.1 GCCTTGGGAGGAGAGGGGTACCTCAATTTCATGGGTAACGAGTTTGGCCATCCTGAGTGG 1860

SBE1.2 ATTGACTTCCCTAGAGAGGGCAATAATTGGTGTTATGACAAATGTAGACGCCAGTGGAAC 1920

SBE1.1 ATTGACTTCCCTAGAGAGGGCAATAATTGGAGTTATGACAAATGTAGACGCCAGTGGAAC 1920

SBE1.2 CTTGCGGATAGCGAACACTTGAGATACAAGTTTATGAATGCATTTGATAGAGCTATGAAT 1980

SBE1.1 CTCGCGGATAGCGAACACTTGAGATACAAGTTTATGAATGCATTTGATAGAGCTATGAAT 1980

SBE1.2 TCGCTCGATGAAAAGTTCTCATTCCTCGCATCAGGAAAACAGATAGTAAGCAGCATGGAT 2040

SBE1.1 TCGCTCGATGAAAAGTTCTCATTCCTCGCATCAGGAAAACAGATAGTAAGCAGCATGGAT 2040

SBE1.2 GATGAGAAGAAGGTTGTTGTGTTTGAACGTGGTGACCTGGTATTTGTATTCAACTTCCAC 2100

SBE1.1 GATGATAATAAGGTTGTTGTGTTTGAACGTGGTGACCTGGTATTTGTATTCAACTTCCAC 2100

SBE1.2 CCAAATAACACATACGAAGGGTATAAAGTTGGATGTGACTTGCCAGGGAAGTACAGAGTT 2160

SBE1.1 CCAAAGAACACATACGAAGGGTATAAAGTTGGATGTGACTTGCCAGGGAAGTACAGAGTT 2160

SBE1.2 GCACTGGACAGTGATGCTTGGGAATTTGGTGGCCATGGAAGAGCTGGTCATGATGTTGAC 2220

SBE1.1 GCACTGGACAGTGATGCTTGGGAATTTGGTGGCCATGGAAGAACTGGTCATGATGTTGAC 2220

SBE1.2 CATTTCACATCACCAGAAGGAATACCTGGAGTTCCAGAAACAAATTTCAATGGTCGTCCA 2280

SBE1.1 CATTTCACATCACCAGAAGGAATACCTGGAGTTCCAGAAACAAATTTCAATGGTCGTCCA 2280

SBE1.2 AATTCCTTCAAAGTGCTGTCTCCTGCGCGAACATGTGTGGCTTATTACAGAGTTGACGAA 2340

SBE1.1 AATTCCTTCAAAGTGCTGTCTCCTGCGCGAACATGTGTGGCTTATTACAGAGTTGATGAA 2340

SBE1.2 CGCATGTCAGAAACTGAAGTTTACCAGACAGACATTTCTAGTGAGCTACTACCAACAGCC 2400

SBE1.1 CGCATGTCAGAAACTGAAGATTACCAGACAGACATTTGTAGTGAGCTACTACCAACAGCC 2400

SBE1.2 AATATCGAGGAGAGTGACGAGAAACTTAAAGATTCGTTATCTACAAATATCAGTAACGTT 2460

SBE1.1 AATATCGAGGAGAGTGACGAGAAACTTAAAGATTCGTTATCTACAAATATCAGTAACATT 2460

SBE1.2 GACGAACTCATGTCAGAAACTGAAGTTTACCAGACAGACATTTCTAGTGAGCTACTACCA 2520

SBE1.1 GACGAACGCATGTCAGAAACTGAAGTTTACCAGACAGACATTTCTAGTGAGCTACTACCA 2520

SBE1.2 ACAGCCAGTATCGAGGAGAGTGACGAGAAACTTAAAGATTCATTATCTACAAATATCAGT 2580

SBE1.1 ACAGCCAATATTGAGGAGAGTGACGAGAAACTTAAAGATTCGTTATCTACAAATATCAGT 2580

SBE1.2 AACATTGGTCAGACTGTTGTAGTTTCTGTTGAGGAGAGAGACAAGGAACTTAAAGATTCA 2640

SBE1.1 AACATTGATCAGACTGTTGTAGTTTCTGTTGAGGAGAGAGACAAGGAACTTAAAGATTCA 2640

SBE1.2 CCATCTGTAAGCATCATTAGTGATGTTGTTCCAACTGAATGGGATGATTCGGATGCAAAC 2700

SBE1.1 CCGTCTGTAAGCATCATTAGTGATGTTGTTCCAGCTGAATGGGATGATTCAGATGCAAAC 2700

SBE1.2 GTCTGGGGTGAGGACTAG 2718

SBE1.1 GTCTGGGGTGAGGACTAG 2718

**b**

SBE2.2 ATGGTGTATACACTCTCTGGAGTTCGTTTTCCTACTGTTCCATCAGTGTACAAATCTAAT 60

SBE2.1 ATGGTGTATACACTCTCTGGAGTTCGTTTTCCTACTGTTCCATCAGTGTACAAATCTAAT 60

SBE2.2 GGATTCAGCAGTAATGGTGATCGGAGGAATGCTAATATTTCTGTATTCTTGAAAAAACAC 120

SBE2.1 GGATTCAGCAGTAATGGTGATCGGAGGAATGCTAATGTTTCTGTATTCTTGAAAAAGCAC 120

SBE2.2 TCTCTTTCACGGAAGATCTTGGCTGAAAAGTCTTCTTACAATTCCGAATCCCGACCTTCT 180

SBE2.1 TCTCTTTCACGGAAGATCTTGGCTGAAAAGTCTTCTTACAATTCCGAATCCCGACCTTCT 180

SBE2.2 ACAGTTGCAGCATCGGGGAAAGTCCTTGTGCCTGGAACCCAGAGTGATAGCTCCTCATCC 240

SBE2.1 ACAGTTGCAGCATCGGGGAAAGTCCTTGTGCCTGGAACCCAGAGTGATAGCTCCTCATCC 240

SBE2.2 TCAACAGATCAATTTGAGTTCACTGAGACATCTCCAGAAAATTCCCCAGCATCAACTGAT 300

SBE2.1 TCAACAGACCAATTTGAGTTCACTGAGACATCTCCAGAAAATTCCCCAGCATCAACTGAT 300

SBE2.2 GTAGATAGTTCAACAATGGAACACGCTAGCCAGATTAAAACTGAGAACGATGACGTTGAG 360

SBE2.1 GTAGATAGTTCAACAATGGAACACGCTAGCCAGATTAAAACTGAGAACGATGACGTTGAG 360

SBE2.2 CCGTCAAGTGATCTTACAGGAAGTGTTGAAGAGCTGGATTTTGCTTCATCACTACAACTA 420

SBE2.1 CCGTCAAGTGATCTTACAGGAAGTGTTGAAGAGCTGGATTTTGCTTCATCACTACAACTA 420

SBE2.2 CAAGAAGGTGGTAAACTGGAGGAGTCTAAAACATTAAATACTTCTGAAGAGACAATTATT 480

SBE2.1 CAAGAAGGTGGTAAACTGGAGGAGTCTAAAACATTAAATACTTCTGAAGAGACAATTATT 480

SBE2.2 GATGAATCTGATAGGATCAGAGAGAGGGGCATCCCTCCACCTGGACTTGGTCAGAAGATT 540

SBE2.1 GATGAATCTGATAGGATCAGAGAGAGGGGCATCCCTCCACCTGGACTTGGTCAGAAGATT 540

SBE2.2 TATGAAATAGACCCCCTTTTGACAAACTATCGTCAACACCTTGATTACAGGTATTCACAG 600

SBE2.1 TATGAAATAGACCCCCTTTTGACAAACTATCGTCAACACCTTGATTACAGGTATTCACAG 600

SBE2.2 TACAAGAAACTGAGGGAGGCAATTGACAAGTATGAGGGTGGTTTGGAAGCTTTTTCTCGT 660

SBE2.1 TACAAGAAACTGAGGGAGGCAATTGACAAGTATGAGGGTGGTTTGGAAGCTTTTTCTCGT 660

SBE2.2 GGTTATGAAAAAATGGGTTTCACTCGTAGTGCTACAGGTATCACTTACCGTGAGTGGGCT 720

SBE2.1 GGTTATGAAAAAATGGGTTTCACTCGTAGTGCTACAGGTATCACTTACCGTGAGTGGGCT 720

SBE2.2 CCTGGTGCCCAGTCAGCTGCCCTCATTGGGGATTTCAACAATTGGGACGCAAATGCTGAC 780

SBE2.1 CCTGGTGCCCAGTCAGCTGCCCTCATTGGGGATTTCAACAATTGGGACGCAAATGCTGAC 780

SBE2.2 ATTATGACTCGGAATGAATTTGGTGTCTGGGAGATTTTTCTGCCAAATAATGTGGATGGT 840

SBE2.1 ATTATGACTCGGAATGAATTTGGTGTCTGGGAGATTTTTCTGCCAAATAATGTGGATGGT 840

SBE2.2 TCTCCTGCAATTCCTCATGGGTCCAGAGTGAAGATACGTATGGACACTCCATCAGGTGTT 900

SBE2.1 TCTCCTGCAATTCCTCATGGGTCCAGAGTGAAGATACGTATGGACACTCCATCAGGTGTT 900

SBE2.2 AAGGATTCCATTCCTGCTTGGATCAACTACTCTTTACAGCTTCCTGATGAAATTCCATAT 960

SBE2.1 AAGGATTCCATTCCTGCTTGGATCAACTACTCTTTACAGCTTCCTGATGAAATTCCATAT 960

SBE2.2 AATGGAATATATTATGATCCACCCGAAGAGGAGAGGTATATCTTCCAACACCCACGGCCA 1020

SBE2.1 AATGGAATATATTATGATCCACCCGAAGAGGAGAGGTATATCTTCCAACACCCACGGCCA 1020

SBE2.2 AAGAAACCAAAGTCGCTGAGAATATATGAATCTCATATTGGAATGAGTAGTCCGGAGCCT 1080

SBE2.1 AAGAAACCAAAGTCGCTGAGAATATATGAATCTCATATTGGAATGAGTAGTCCGGAGCCT 1080

SBE2.2 AAAATTAACTCATACGTGAATTTTAGAGATGAAGTTCTTCCTCGCATAAAAAAGCTTGGG 1140

SBE2.1 AAAATTAACTCATACGTGAATTTTAGAGATGAAGTTCTTCCTCGCATAAAAAAGCTTGGG 1140

SBE2.2 TACAATGCGGTGCAAATTATGGCTATTCAAGAGCATTCTTATTATGCTAGTTTTGGTTAT 1200

SBE2.1 TACAATGCGGTGCAAATTATGGCTATTCAAGAGCATTCTTATTATGCTAGTTTTGGTTAT 1200

SBE2.2 CATGTCACAAATTTTTTTGCACCAAGCAGCCGTTTTGGAACGCCCGACGACCTTAAGTCT 1260

SBE2.1 CATGTCACAAATTTTTTTGCACCAAGCAGCCGTTTTGGAACGCCCGACGACCTTAAGTCT 1260

SBE2.2 TTGATTGATAAAGCTCATGAGCTAGGAATTGTTGTTCTCATGGACATTGTTCACAGCCAT 1320

SBE2.1 TTGATTGATAAAGCTCATGAGCTAGGAATTGTTGTTCTCATGGACATTGTTCACAGCCAT 1320

SBE2.2 GCATCAAATAATACTTTAGATGGACTGAACATGTTTGACGGCACAGATAGTTGTTACTTT 1380

SBE2.1 GCATCAAATAATACTTTAGATGGACTGAACATGTTTGACGGCACAGATAGTTGTTACTTT 1380

SBE2.2 CACTCTGGAGCTCGTGGTTATCATTGGATGTGGGATTCCCGCCTCTTTAACTATGGAAAC 1440

SBE2.1 CACTCTGGAGCTCGTGGTTATCATTGGATGTGGGATTCCCGCCTCTTTAACTATGGAAAC 1440

SBE2.2 TGGGAGGTACTTAGGTATCTTCTCTCAAATGCGAGATGGTGGTTGGATGAGTTCAAATTT 1500

SBE2.1 TGGGAGGTACTTAGGTATCTTCTCTCAAATGCGAGATGGTGGTTGGATGAGTTCAAATTT 1500

SBE2.2 GATGGATTTAGATTTGATGGTGTGACATCAATGATGTATACTCACCACGGATTATCGGTG 1560

SBE2.1 GATGGATTTAGATTTGATGGTGTGACATCAATGATGTATACTCACCACGGATTATCGGTG 1560

SBE2.2 GGATTCACTGGGAACTACGAGGAATACTTTGGACTCGCAACTGATGTGGATGCTGTTGTG 1620

SBE2.1 GGATTCACTGGGAACTACGAGGAATACTTTGGACTCGCAACTGATGTGGATGCTGTTGTG 1620

SBE2.2 TATCTGATGCTGGTCAACGATCTTATTCATGGGCTTTTCCCAGATGCAATTACCATTGGT 1680

SBE2.1 TATCTGATGCTGGTCAACGATCTTATTCATGGGCTTTTCCCAGATGCAATTACCATTGGT 1680

SBE2.2 GAAGATGTTAGCGGAATGCCGACATTTTGTATTCCCGTTCAAGATGGGGGTGTTGGCTTT 1740

SBE2.1 GAAGATGTTAGCGGAATGCCGACATTTTGTATTCCCGTTCAAGATGGGGGTGTTGGCTTT 1740

SBE2.2 GACTATCGGCTGCATATGGCAATTGCTGATAAATGGATTGAGTTGCTCAAGAAACGGGAT 1800

SBE2.1 GACTATCGGCTGCATATGGCAATTGCTGATAAATGGATTGAGTTGCTCAAGAAACGGGAT 1800

SBE2.2 GAGGATTGGAGAGTGGGTGATATTGTTCATACACTGACAAATAGAAGATGGTCGGAAAAG 1860

SBE2.1 GAGGATTGGAGAGTGGGTGATATTGTTCATACACTGACAAATAGAAGATGGTCGGAAAAG 1860

SBE2.2 TGTGTTTCATACGCTGAAAGTCATGATCAAGCTCTAGTCGGTGATAAAACTATAGCATTC 1920

SBE2.1 TGTGTTTCATACGCTGAAAGTCATGATCAAGCTCTAGTCGGTGATAAAACTATAGCATTC 1920

SBE2.2 TGGCTGATGGACAAGGATATGTATGATTTTATGGCTCTGGATAGACCGTCAACATCATTA 1980

SBE2.1 TGGCTGATGGACAAGGATATGTATGATTTTATGGCTTTGGATAGACCGTCAACATCATTA 1980

SBE2.2 ATAGATCGTGGGATAGCATTGCACAAGATGATTAGGCTTGTAACTATGGGATTAGGAGGA 2040

SBE2.1 ATAGATCGTGGGATAGCATTGCACAAGATGATTAGGCTTGTAACTATGGGATTAGGAGGA 2040

SBE2.2 GAAGGGTACCTAAATTTCATGGGAAATGAATTCGGCCACCCTGAGTGGATTGATTTCCCT 2100

SBE2.1 GAAGGGTACCTAAATTTCATGGGAAATGAATTCGGCCACCCTGAGTGGATTGATTTCCCT 2100

SBE2.2 AGGGCTGAACAACACCTCTCTGATGGCTCAGTAATTCCCGGAAACCAATTCAGTTATGAT 2160

SBE2.1 AGGGCTGAACAACACCTCTCTGATGGCTCAGTAATTCCCGGAAACCAATTCAGTTATGAT 2160

SBE2.2 AAATGCAGACGGAGATTTGACCTGGGAGATGCAGAATATTTAAGATACCGTGGGTTGCAA 2220

SBE2.1 AAATGCAGACGGAGATTTGACCTGGGAGATGCAGAATATTTAAGATACCGTGGGTTGCAA 2220

SBE2.2 GAATTTGACCGGGCTATGCAGTATCTTGAAGATAAATATGAGTTTATGACTTCAGAACAC 2280

SBE2.1 GAATTTGACCGGGCTATGCAGTATCTTGAAGATAAATATGAGTTTATGACTTCAGAACAC 2280

SBE2.2 CAGTTCATATCACGAAAGGATGAAGGAGATAGGATGATTGTATTTGAAAAAGGAAACCTA 2340

SBE2.1 CAGTTCATATCACGAAAGGATGAAGGAGATAGGATGATTGTATTTGAAAAAGGAAACCTA 2340

SBE2.2 GTTTTTGTCTTTAATTTTCACTGGACAAAAAGCTATTCAGACTATCGCATAGGCTGCCTG 2400

SBE2.1 GTTTTTGTCTTTAATTTTCACTGGACAAAAAGCTATTCAGACTATCGCATAGGCTGCCTG 2400

SBE2.2 AAGCCTGGAAAATACAAGGTTGCCTTGGACTCAGATGATCCACTTTTTGGTGGCTTCGGG 2460

SBE2.1 AAGCCTGGAAAATACAAGGTTGCCTTGGACTCAGATGATCCACTTTTTGGTGGCTTCGGG 2460

SBE2.2 AGAATTGATCATAATGCCGAATATTTCACCTTTGAAGGATGGTATGATGATCGTCCTCGT 2520

SBE2.1 AGAATTGATCATAATGCCGAATGTTTCACCTTTGAAGGATGGTATGATGATCGTCCTCGT 2520

SBE2.2 TCAATTATGGTGTATGCACCTAGTAGAACAGCAGTGGTCTATGCACTAGTAGACAAAGAA 2580

SBE2.1 TCAATTATGGTGTATGCACCTAGTAGAACAGCAGTGGTCTATGCACTAGTAGACAAAGAA 2580

SBE2.2 GAAGAAGAAGAAGAAGAAGTAGCAGTAGTAGAAGAAGTAGTAGTAGAAGAAGAATGA 2637

SBE2.1 GAAGAAGAAGAAGTAGCAGTAGTAGAAGAAGT------AGTAGTAGAAGAAGAATGA 2637

**Figure S3.** Design of constructs for RNA-guided Cas9-mediated mutagenesis of *SBE* genes.


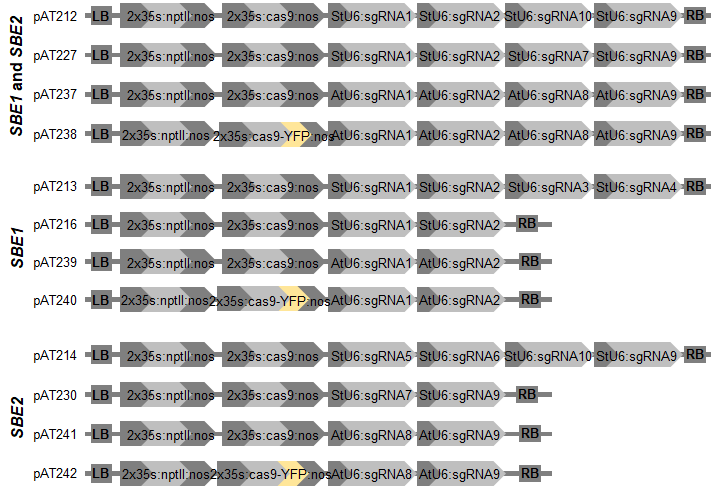
Constructs contained combinations of the sgRNAs specified in Figure S1 with a selectable marker gene and the Cas9 coding sequence driven by the 2x35S promoter. Constructs pAT212, pAT227, pAT213, pAT216, pAT214 and pAT230 were used for *Agrobacterium*-mediated transformation. Each sgRNA was driven by the potato U6 promoter. Constructs pAT237, pAT238, pAT239, pAT240, pAT241 and pAT242 were used for PEG-mediated transformation of protoplasts. Each sgRNA was driven by the Arabidopsis U6 promoter. Constructs 238, 240 and 242 contained a Cas9:YFP fusion protein, and were introduced into protoplasts in order to monitor the efficiency of delivery. LB = left border; RB = right border.

**Figure S4.** PCR analysis of *SBE1* in a selection of lines generated by **a**, *Agrobacterium*-mediated transformation and **b**, PEG-mediated protoplast transformation.

Primers (oAT138-F and oAT123-R) span the target sites recognised by sgRNA1 and sgRNA2 amplifying a 606 bp fragment in wild-type plants. The presence of smaller band(s) seen in many lines indicates the deletion of sequence between the targets. Genotype designations a, b etc. indicate plants derived from different shoots from a single callus. Lines from which starch was analysed are highlighted in yellow. MW: molecular weight marker (100 bp DNA ladder, New England Biolabs). WT: wild type DNA from a non-transformed plant regenerated in tissue culture. NC: No-template PCR control.


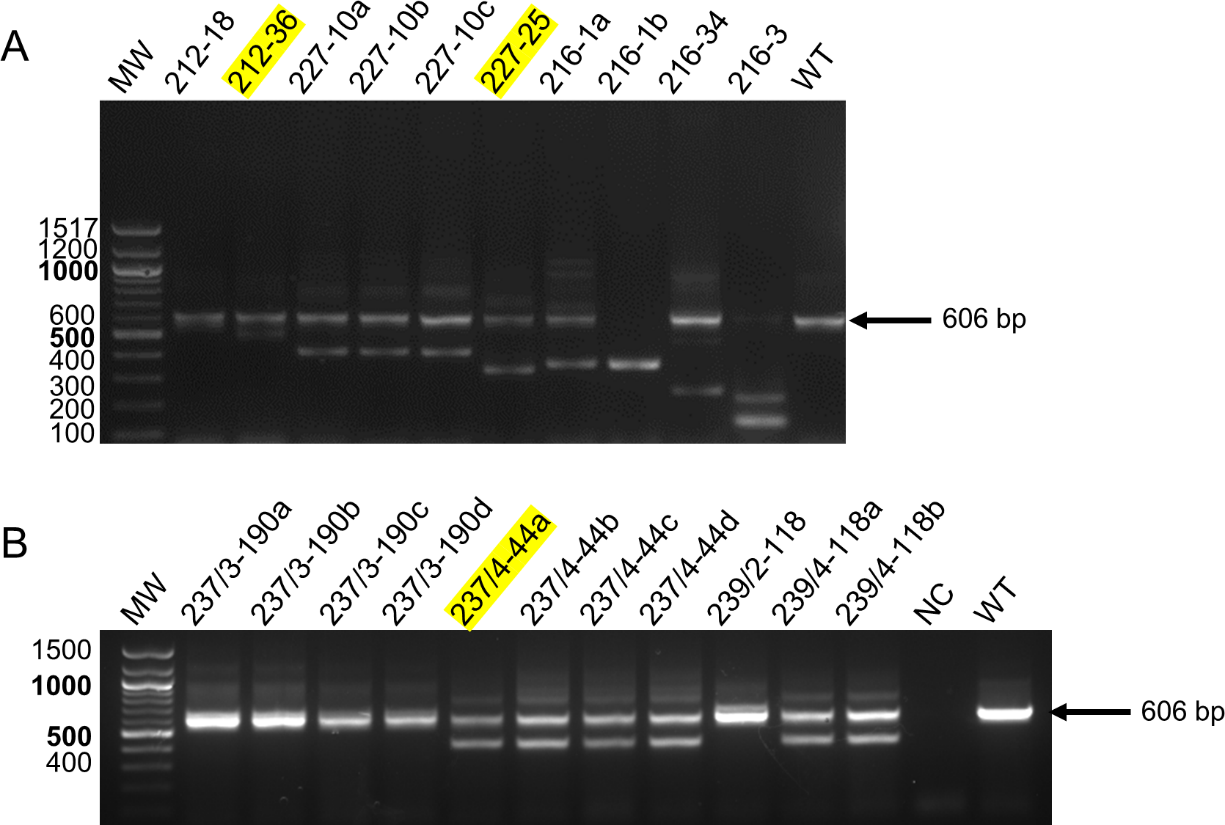


**Figure S5.** PCR analysis of *SBE2* in a selection of lines generated by **a**, *Agrobacterium*-mediated transformation and **b**, PEG-mediated protoplast transformation

Primers span the target sites recognised by sgRNAs 9 and 10 (oAT184-F and oAT244-R), 7 and 9 (oAT55-F and oAT244-R) and 8 and 9 (oAT134-F and oAT244-R). The presence of smaller band(s) seen in many lines indicates the deletion of sequence between the targets. Genotype designations a, b etc. indicate plants derived from different shoots from a single callus. Lines from which starch was analysed are highlighted in yellow. MW: molecular weight marker (100 bp DNA ladder, New England Biolabs). WT: wild type DNA from a non-transformed plant regenerated in tissue culture. NC: No-template PCR control.

**
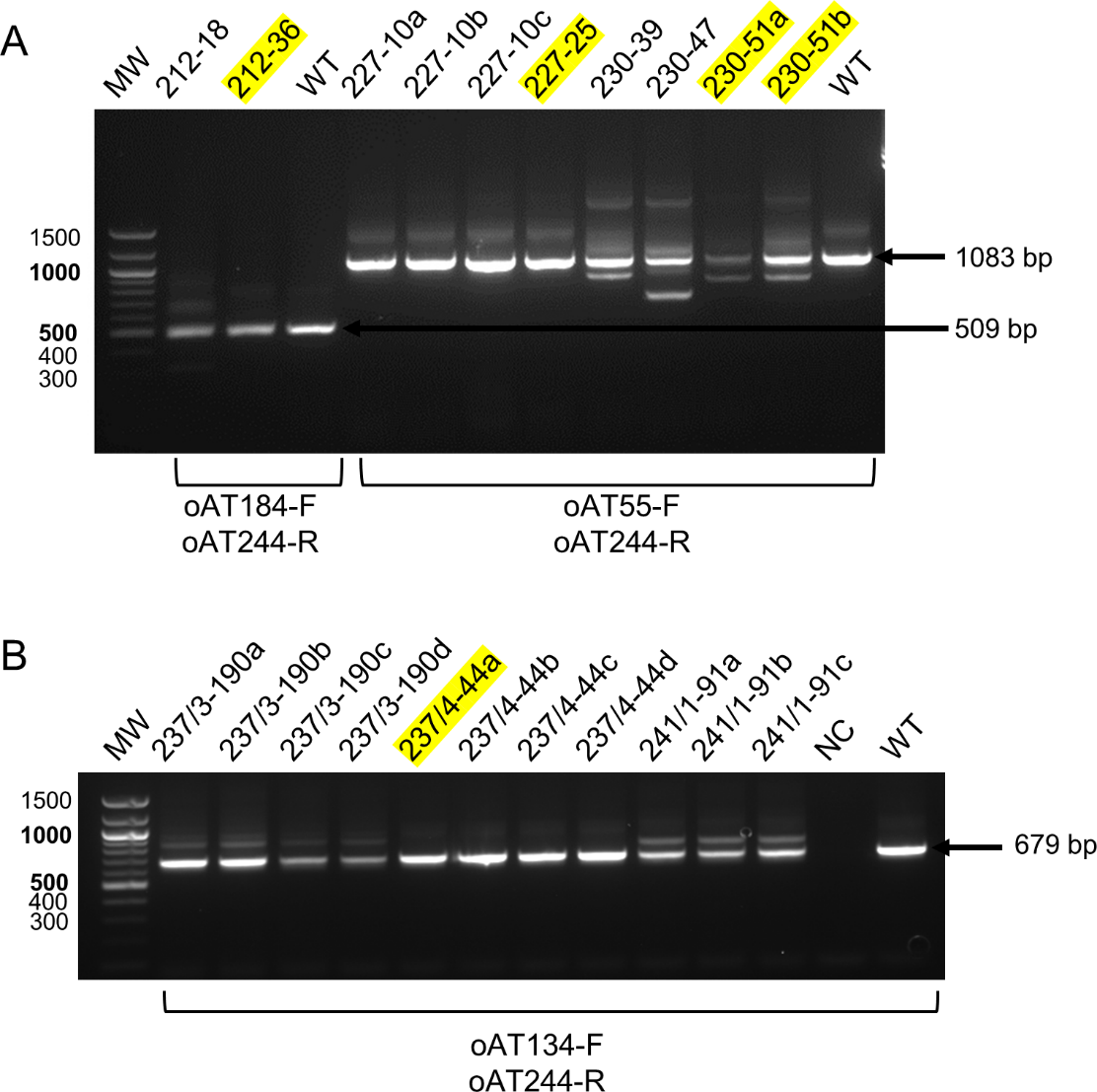
**

**Figure S6.** Examples of confirmation of Cas9 expression and mutation of *SBE* genes in protoplasts three days after PEG-mediated protoplast transformation.

**a**, Protoplasts transformed with the plasmid pAT238. YFP (bright green) is present throughout some protoplasts and confined to nuclei in many others. Scale bar represents 100 µm. **b**, Agarose gel electrophoresis of PCR amplicons amplified using gDNA extracted from samples of protoplasts transformed with (pAT240, pAT242) or without (pAT239, pAT241) constructs encoding a Cas9-YFP fusion. Primers span the target sites recognised by sgRNAs 1 and 2 (oAT138-F and oAT148-R) or 8 and 9 (oAT134-F and oAT244-R). The presence of smaller band(s) seen in the transformed samples indicate the deletion of sequence between the targets in some cells. Lanes 1 and 2 are from two independent batches of protoplasts for each construct. Control: protoplasts mock-transformed with water instead of plasmid DNA. MW: Molecular weight marker (100 bp DNA ladder, New England Biolabs).

689 bp

679 bp


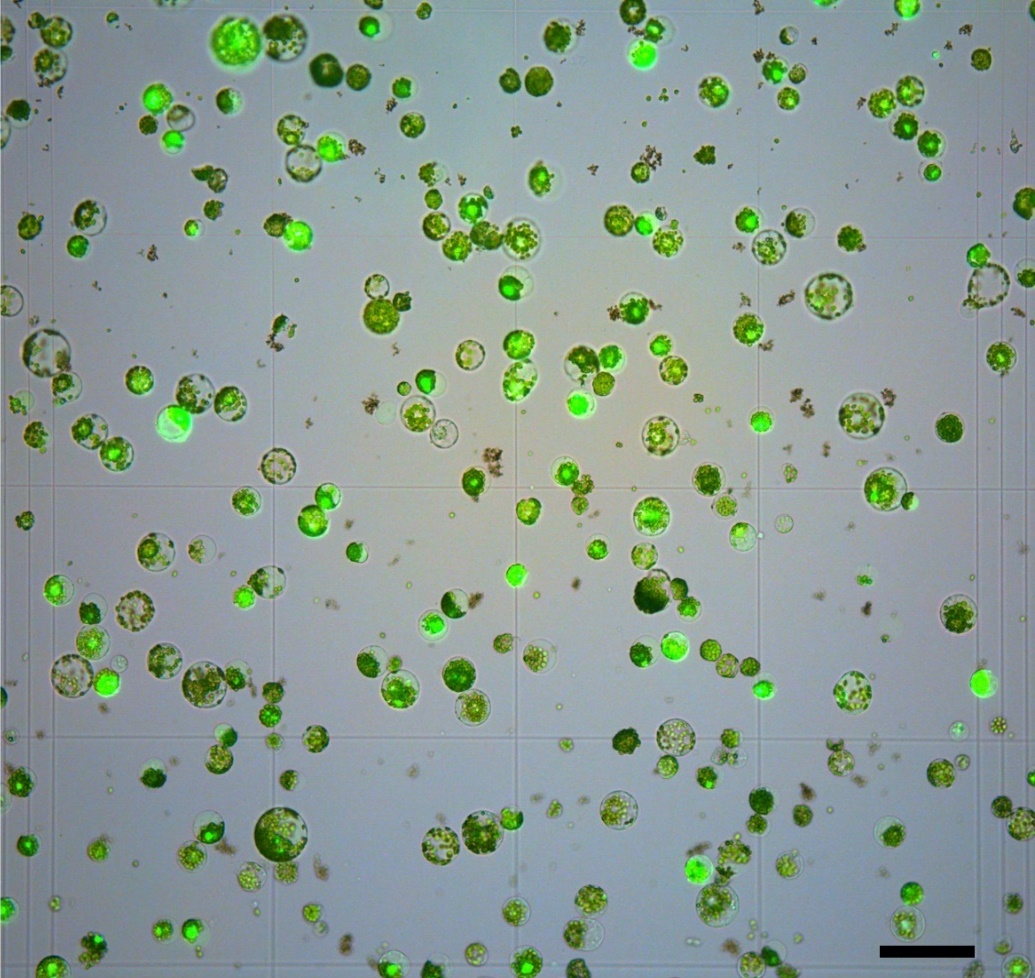

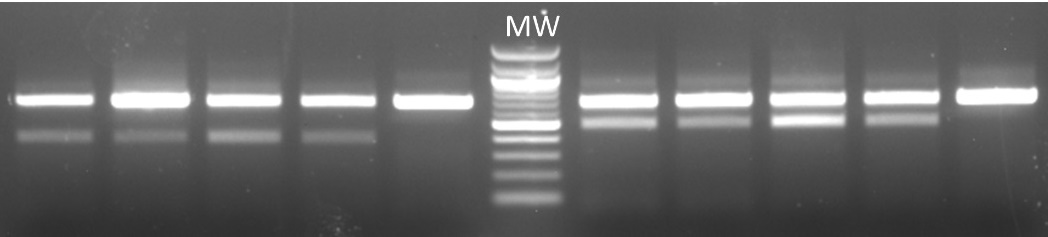


pAT239

pAT240

pAT239

pAT240

pAT241

pAT242

pAT241

pAT242

control

control

1 2 1 2

*SBE1*

*SBE2*

a

b

**Figure S7.** Detection of Cas9 transgene in protoplast-derived plants.

Primers (oAT168-F and oAT169R) were used to amplify ~1 kb region of the Cas9 gene from genomic DNA purified from rooted plantlets prior to transplantation into soil. Each lane represents a single plant. Where there are multiple lanes with the same label individual plants were derived from the same callus. The Cas9 transgene was detected in only 8 out of 25 plants. The negative control (wild-type) plant was regenerated from protoplasts mock-transformed with water. The positive control (PC) is gDNA of the mutant line 212-36, generated by *Agrobacterium*-mediated transformation. The no-template control (NC) did not contain DNA in the PCR. MW: Molecular weight marker (1 kb ladder, New England Biolabs).

Wild-type

NC PC

237/0-115


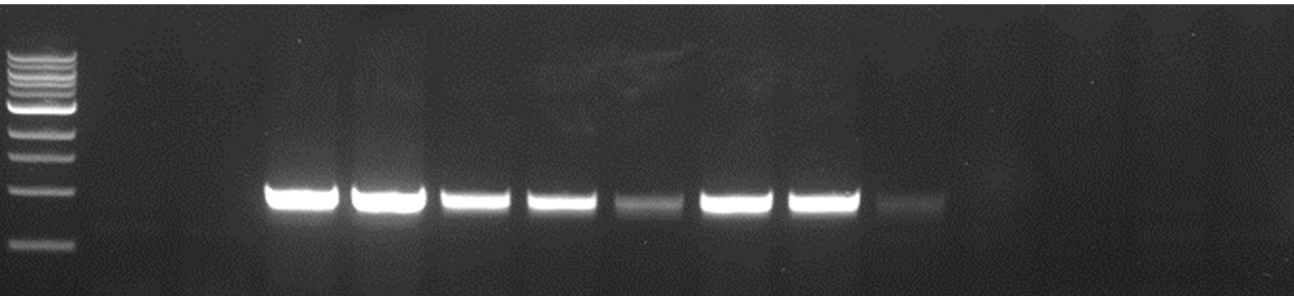


237/0-199

237/3-42

237/3-46

237/3-138

237/3-190

MW

239/2-118

237/3-190

237/4-44

239/4-118

241/1-91

MW


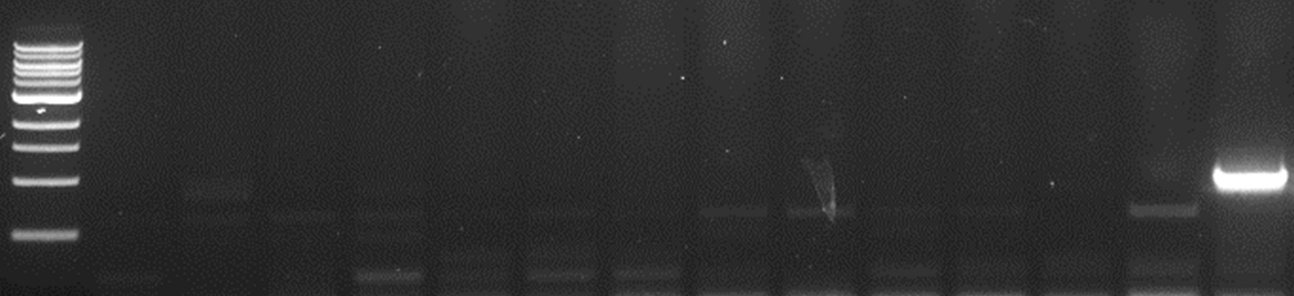


3000

2000

1500

1000

500

3000

2000

1500

1000

500

**Figure S8.** Sequence analysis of *SBE1* from line 237/4-44. Direct sequencing of PCR amplicons and sequencing of cloned amplicons provided no evidence of wild type sequence and evidence of three different repair events: (i) a two base pair deletion (ii) a six base-pair deletion and (iii) a large (189 bp) deletion of the DNA between two sgRNAs. These data suggest that *SBE1.1* may have a homozygous mutation and *SBE1.2* a biallelic mutation. Line 237/4-44 was regenerated from protoplasts and does not contain a Cas9 transgene. Exons are shaded in grey and target sequences of sgRNAs are shown in bold letters. Long stretches of sequence are indicated by dots (“…”); short nucleotide deletions by dashes, “-“; longer deletions by continuous lines, “_”. MW: Molecular weight marker (100 bp DNA ladder, New England Biolabs).


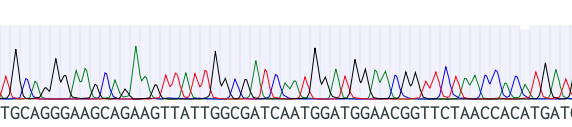

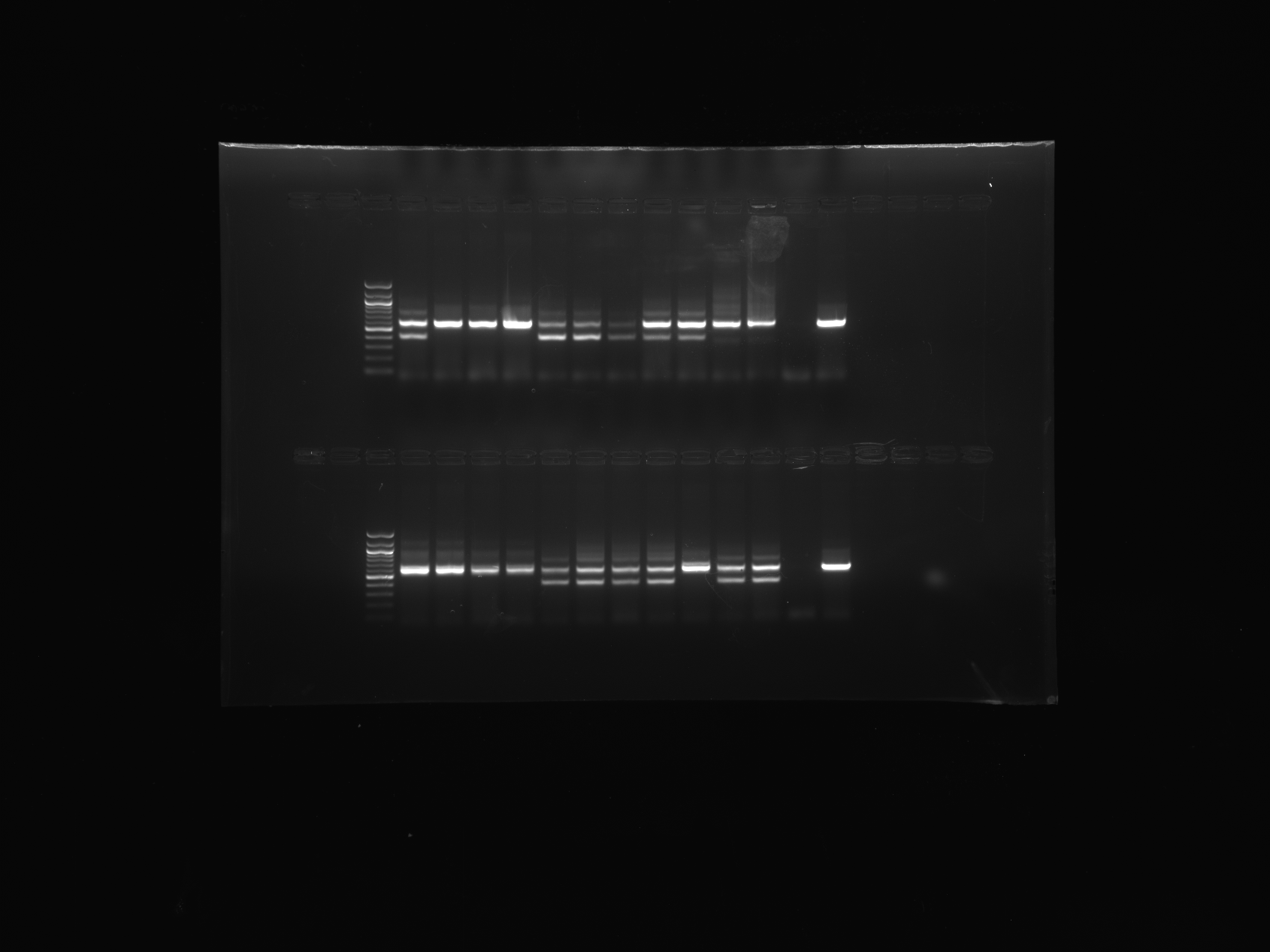


**sgRNA-1**

...CAGGTTATTTAAAATTTGGATTCAACAGGGAAGATG**GTTGCATAGTCTATCGTGAA**TGGGCTCCTGCTGCTCAGTA......CAGGGAAGCA**GAAGTTATTGGCGATTTCAA**TGG...GAGTT...

...CAGGTTATTTAAAATTTGGATTCAACAGGGAAGATG**GTTGCATAGTCTATCGTGAA**TGGGCTCCTGCTGCTCAGTA......CAGGGAAGCA**GAAGTTATTGGCGAT--CAA**TGG...GAGTT...

...CAGGTTATTTAAAATTTGGATTCAACAGGGAAGATG**GTTGCATAGTCTATCGTGAA**TGGGCTCCTGCTGCTCAGTA......CAGGGAAGCA**GAAGTTATTGG------CAA**TGG...GAGTT...

...CAGGTTATTTAAAATTTGGATTCAACAGGGAAGATG**GTTGCATAGTCTATCGT**_______________________________________**_________________CAA**TGG...GAGTT...

**sgRNA-2**

Direct sequencing of the larger amplicon obtained using primers oAT138-F and oAT123-R flanking the target sites for sgRNA1 and sgRNA2 showed double peaks following the target site for sgRNA2 indicating the presence of multiple sequence variants. Cloning of this amplicon followed by sequencing of six colonies indicated the presence of two repair events, a two base pair deletion and a six base pair deletion.
No wild type sequence was observed.

For full details of gel
see Figure S3

189 bp deleted

**2 base pair deletion**

‘T ‘at this position indicates this sequence is from *SBE1.1*

Primers: oAT138-F and oAT123-R

**1000**

1500

400

**500**

WT

NC

239/4-118b

239/4-118a

239/2-118

237/4-44d

237/4-44c

237/4-44a

237/4-44b

237/3-190d

237/3-190c

237/3-190b

237/3-190a

MW

**
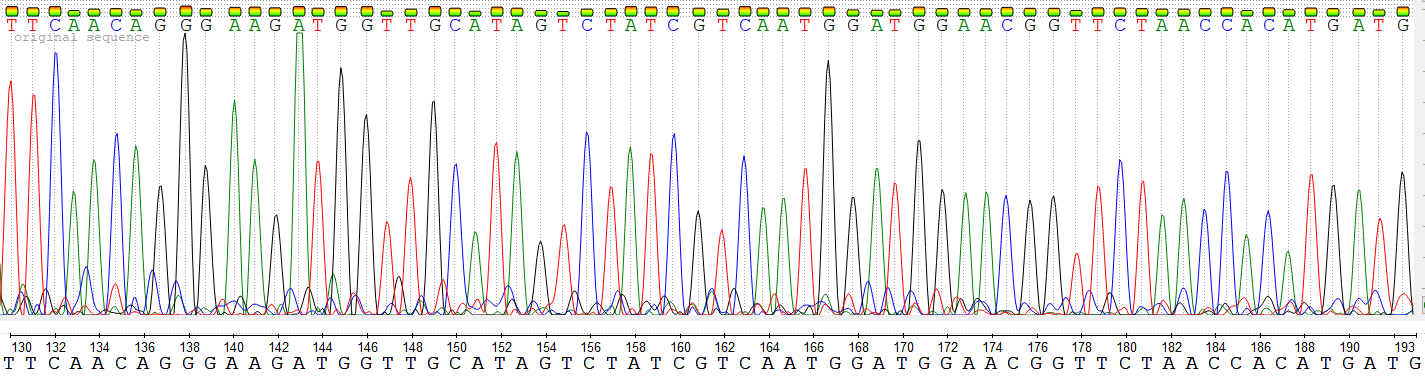
**
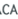

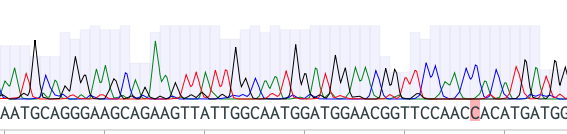


**6 base pair deletion**

606 bp

‘T’ at this position indicates this sequence is from *SBE1.1*

**189 bp deletion**

**sgRNA1**

**sgRNA 2**

Direct sequencing of the smaller amplicon obtained using primers oAT138-F and
oAT123-R flanking the target sites for sgRNA1 and sgRNA2 indicates the deletion of 189 bp
between the targets:

‘C’ at this position indicates this sequence is from *SBE1.2*

WT

237/4-44

237/4-44

237/4-44

**Figure S9.** Sequence analysis of *SBE2* from line 237/4-44.

Direct sequencing of amplicons and sequencing of cloned amplicons provided no evidence of wild type sequence and evidence of three different repair events (i) a one base pair deletion (ii) a two base-pair deletion and (iii) a six base pair deletion. These data suggest that one homoeoallele may have a homozygous mutation and the other a biallelic mutation. Line 237/4-44 was regenerated from protoplasts and does not contain a Cas9 transgene.

Direct sequencing of the PCR amplicon obtained using primers (oAT134-F and oAT244-R) flanking the target sites for sgRNA8 and sgRNA9 showed multiple peaks following the predicted cleavage site within the target for sgRNA 9. This indicates the presence of multiple sequence variants. The absence of wild-type sequence can be inferred by the lack of a G at position 196 where multiple peaks start to appear.


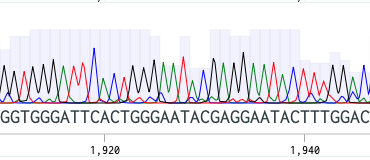

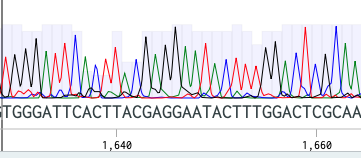

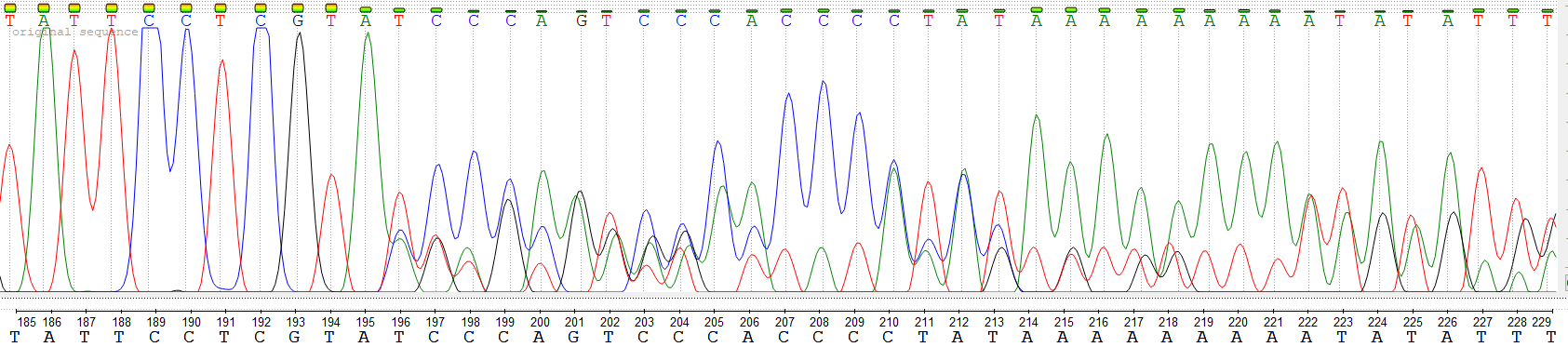

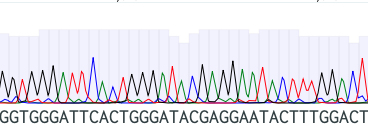


6 base pair deletion

2 base pair deletion

1 base pair deletion

**sgRNA-9 (reverse complement)**

**sgRNA-9**

TAATTTCTATAGGT**GGGATTCACTGGGAACTACG**AGGAATACTTTGGACTCGCAACTGATGTGGATGCTG

TAATTTCTATAGGT**GGGATTCACTGGGAA-TACG**AGGAATACTTTGGACTCGCAACTGATGTGGATGCTG

TAATTTCTATAGGT**GGGATTCACTGGGA--TACG**AGGAATACTTTGGACTCGCAACTGATGTGGATGCTG

TAATTTCTATAGGT**GGGATTCACT------TACG**AGGAATACTTTGGACTCGCAACTGATGTGGATGCTG

TATTCCTCGTAGTTCCCAGTGAATCCCACCTATAGAAATTAAAA

Wild type:

direction of sequencing

WT

237/4-44

237/4-44

237/4-44

237/4-44:

Cloning of this amplicon followed by sequencing of multiple colonies indicate the presence of three different repair events.

**Figure S10.** Sequence analysis of *SBE2* from line 230-51a and b.

Direct sequencing of amplicons and sequencing of cloned amplicons provided evidence of three different repair events. No wild type sequence was observed. Exons are shaded in grey and target sequences of sgRNAs are shown in bold letters. Long stretches of sequence are indicated by dots (“…”); short nucleotide deletions by dashes, “-“; longer deletions by continuous lines, “_”.


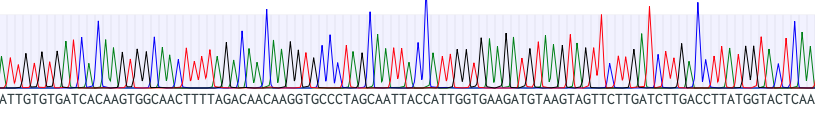

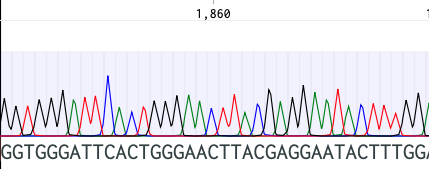

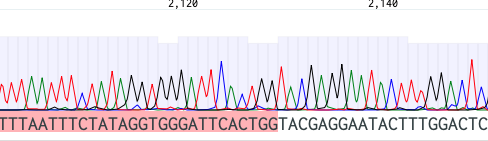

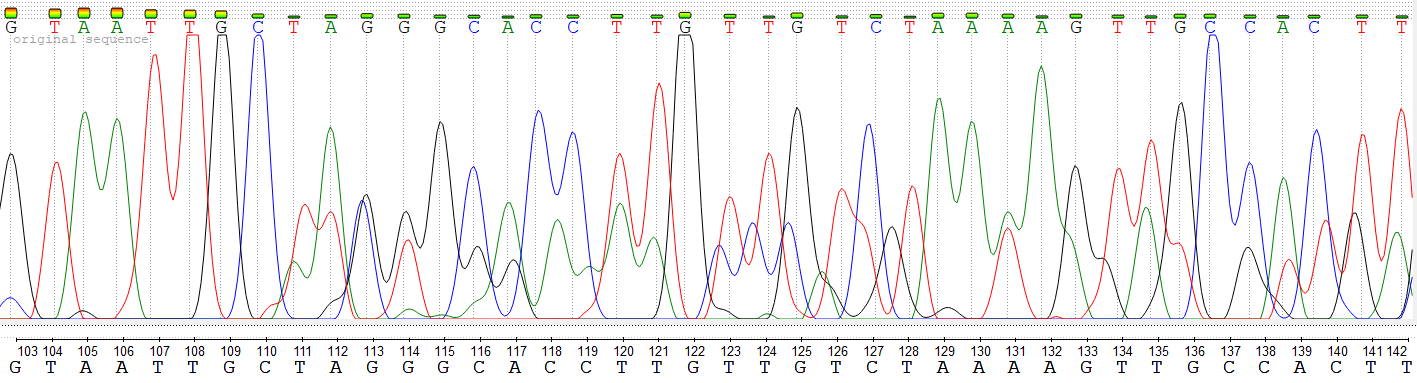


Direct sequencing of the amplicons obtained using primers (oAT55-F and oAT244-R) flanking the target sites for sgRNA 7 and sgRNA9 provided evidence of multiple peaks starting before the recognition site for sgRNA9. This indicates the presence of multiple sequences including a deletion. The same result was obtained from plant 230-51b.

... AGACAACAAGGTGCCCTAG...TAATTTCTATAGGT**GGGATTCACTGGGAAC TACG**AGGAATACTTTGGACTCGCAACTGATGTGGATGCTG...CAATTACCATTGGTGAAGATGTAAGTAG...

... AGACAACAAGGTGCCCTAG...TAATTTCTATAGGT**GGGATTCACTGGGAACTTACG**AGGAATACTTTGGACTCGCAACTGATGTGGATGCTG...CAATTACCATTGGTGAAGATGTAAGTAG...

... AGACAACAAGGTGCCCTAG...TAATTTCTATAGGT**GGGATTCACTGG---- TACG**AGGAATACTTTGGACTCGCAACTGATGTGGATGCTG...CAATTACCATTGGTGAAGATGTAAGTAG...

... AGACAACAAGGTGCCCTAG_________________**____________________**________________________________________CAATTACCATTGGTGAAGATGTAAGTAG...

**sgRNA9**

1 base pair insertion

4 base pair deletion

214 bp deletion

TAATTTCTATAGGTGGGATTCACTGGTACGAGGAATACTTTGGACTC

**Gtaattgcatctggg**aaaagcccatgaataagatcgttgaccagcat

Wild type (reverse complement):

230-51a:

Following cloning, sequencing of fourteen colonies (seven from plant 230-51a and seven from 230-51b) provided evidence of the same three different repair events in both plants: (i) a 1 bp insertion, ii) a 4 bp deletion and (iii) a 214 bp deletion that correlated with start of the multiple peaks observed by direct sequencing of the PCR amplicon. No wild type sequence was observed.

**Figure S11.** Sequence analysis of *SBE1* from line 227-25.

Direct sequencing of amplicons and sequencing of cloned amplicons provided evidence of wild type sequence and one repair event. Exons are shaded in grey and target sequences of sgRNAs are shown in bold letters. Long stretches of sequence are indicated by dots (“…”); short nucleotide deletions by dashes, “-“; longer deletions by continuous lines, “_”.


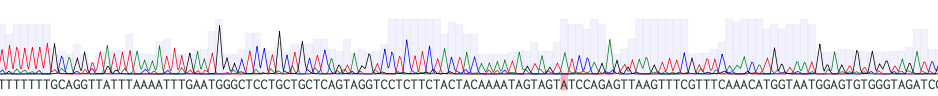

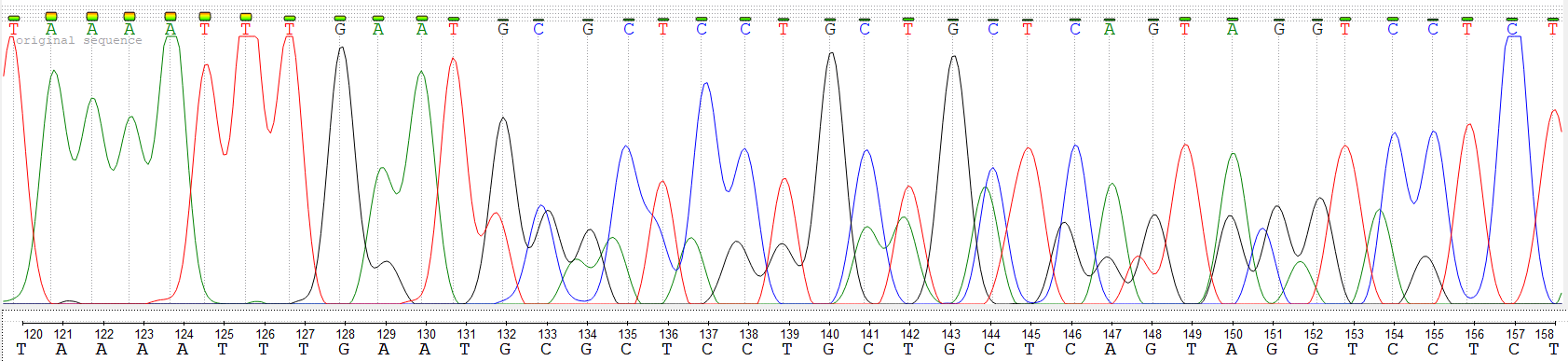


Direct sequencing of the amplicons obtained using primers (oAT138-F and oAT123-R) flanking the target sites for sgRNA1 and sgRNA2 showed double peaks close to the target site for sgRNA1 indicating the presence of two sequence variants:

**sgRNA-1**

TAAAATTTGGATTCAACAGGGAAGATGGTTGCATAGTCTAG

Following cloning, sequencing of six colonies confirmed the presence of (i) wild type sequence and (ii) a sequence
with two deletions:

...CAGGTTATTTAAAATTTGGATTCAACAGGGAAGATG**GTTGCATAGTCTATCGTGAA**TGGGCTCCTGCTGCTCAGTAggtcctcttctactacaaaatagtagtttccatcat...Gacagtaagccagtcattccacacaactccagagttaagtttcgtttcaaacatggtaa...

...CAGGTTATTTAAAATTTG__________________**__________________AA**TGGGCTCCTGCTGCTCAGTAggtcctcttctactacaaaatagtagt_______________________________________tccagagttaagtttcgtttcaaacatggtaa...

WT

227-25

36 bp deletion

241 bp deletion

Wild type:

227-25:

**Figure S12.** Sequence analysis of *SBE1* from line 212-36.

Direct sequencing of amplicons and sequencing of cloned amplicons provided evidence of wild type sequence and one repair event. Exons are shaded in grey and target sequences of sgRNAs are shown in bold letters. Long stretches of sequence are indicated by dots (“…”); short nucleotide deletions by dashes, “-“; longer deletions by continuous lines, “_”.


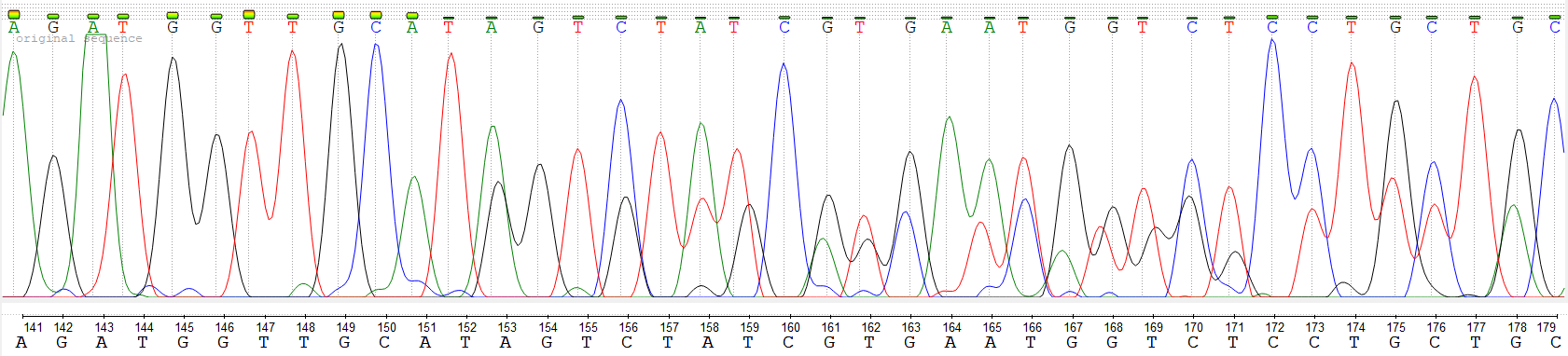

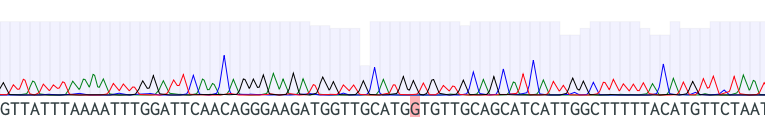


Direct sequencing of the amplicons obtained using primers (oAT138-F and oAT123-R) flanking the target sites for sgRNA1 and sgRNA2 showed double peaks from within the target site for sgRNA1 indicating the presence of two sequence variants:

Following cloning, sequencing of five colonies confirmed the presence of wild type sequence and a sequence with a 95 bp deletion.

...CAGGTTATTTAAAATTTGGATTCAACAGGGAAGATG**GTTGCATAGTCTATCGTGAA**TGGGCTCCTGCTGCTCAgtaggtcctcttctactacaaaatagtagtttccatcatcataacagattttcctattaaagcatgatgttgcagcatcattggcttttt

...CAGGTTATTTAAAATTTGGATTCAACAGGGAAGATG**GTTGCATGGT__________**_____________________________________________________________________________________gttgcagcatcattggcttttt

WT

212-36

AGATGGTTGCATAGTCTATCGTGAATGGGCTCC

**sgRNA1**

95 bp deletion

Wild type:

212-36:

**Figure S13.** Sequence analysis of *SBE2* from line 212-36.

Direct sequencing of amplicons and sequencing of cloned amplicons provided evidence of multiple repair events. Exons are shaded in grey and target sequences of sgRNAs are shown in bold letters. Long stretches of sequence are indicated by dots (“…”); short nucleotide deletions by dashes, “-“; longer deletions by continuous lines, “_”.


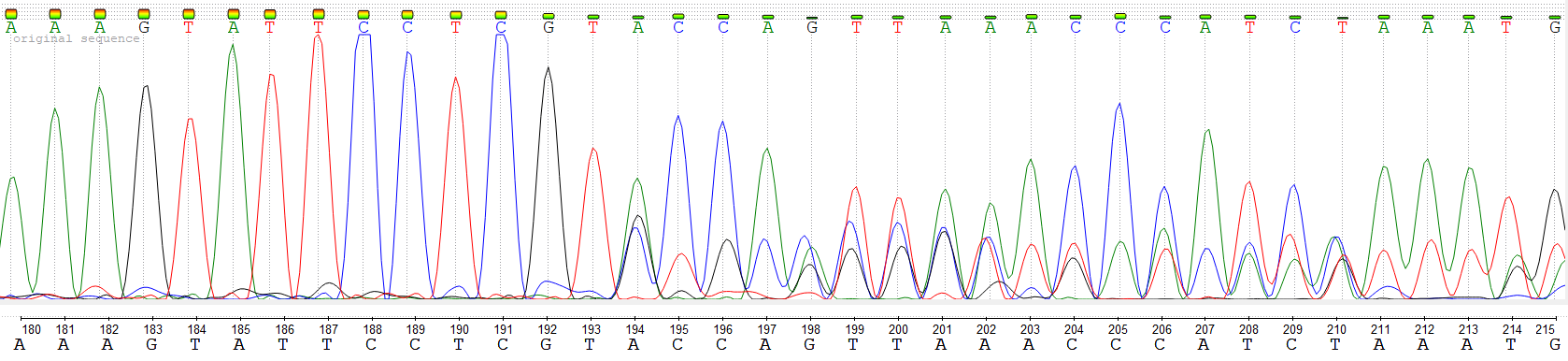

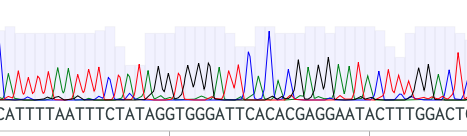

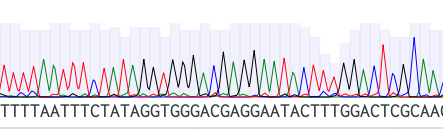


Direct sequencing of the amplicons obtained using primers (oAT184-F and oAT244-R) flanking the target sites for sgRNA9 and sgRNA10 showed multiple peaks from within the target site for sgRNA9 indicating the presence of at least three sequence variants:

Following cloning, sequencing of four colonies confirmed the presence of sequences with (i) an 8 bp deletion and (ii) a 14 bp deletion. However, the data from the direct sequencing suggests the presence of other sequence variants.

**sgRNA-9 (reverse complement)**

AAAGTATTCCTCGTAGTTCCCAGTGAATCCCACCGA

Wild type:

212-36:

... AGACAACAAGGTGCCCTAG...TAATTTCTATAGGT**GGGATTCACTGGGAACTACG**AGGAATACTTTGGACTCGCAACTGATGTGGATGCTG...CAATTACCATTGGTGAAGATGTAAGTAG...

... AGACAACAAGGTGCCCTAG...TAATTTCTATAGGT**GGGATTCAC--------ACG**AGGAATACTTTGGACTCGCAACTGATGTGGATGCTG...CAATTACCATTGGTGAAGATGTAAGTAG...

... AGACAACAAGGTGCCCTAG...TAATTTCTATAGGT**GGG--------------ACG**AGGAATACTTTGGACTCGCAACTGATGTGGATGCTG...CAATTACCATTGGTGAAGATGTAAGTAG...

WT

212-36

212-36

8 bp deletion

14 bp deletion

**Figure S14.** Sequence analysis of *SBE2* from line 227-25.

Direct sequencing of amplicons and sequencing of cloned amplicons provided evidence of wild type sequence and one repair event. Exons are shaded in grey and target sequences of sgRNAs are shown in bold letters. Long stretches of sequence are indicated by dots (“…”); short nucleotide deletions by dashes, “-“; longer deletions by continuous lines, “_”.


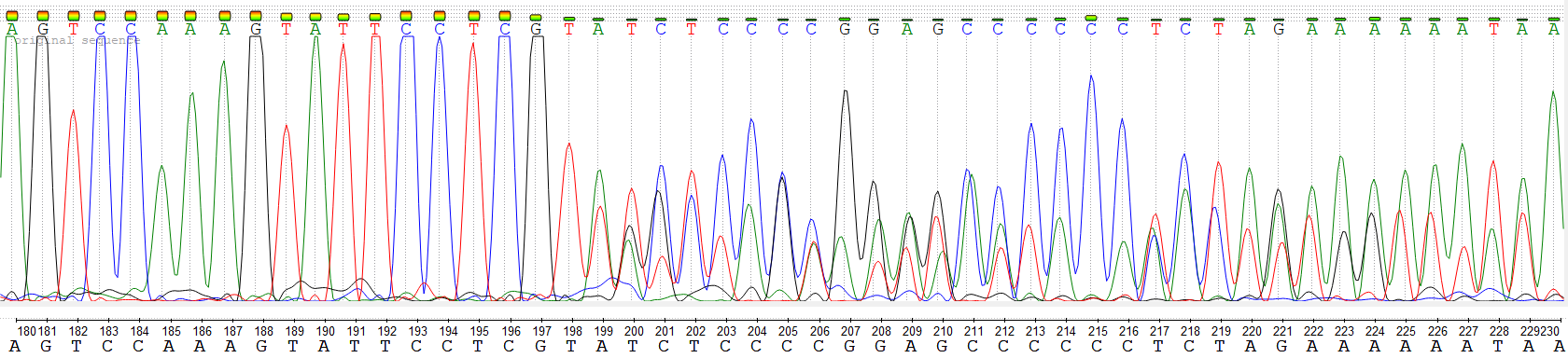


**sgRNA-9 (reverse complement)**

TATTCCTCGTAGTTCCCAGTGAATCCCACCTATAGAAATTAAAA

Wild type:

direction of sequencing

Direct sequencing of the amplicons obtained using primers (oAT55-F and oAT244-R) flanking the target sites for sgRNA7 and sgRNA9 showed triple peaks close to the predicted cleavage site of sgRNA9 indicating the presence of at least two sequence variants:

Following cloning, sequencing of six colonies confirmed the presence of wild type sequence and sequence with a 1bp insertion.

... AGACAACAAGGTGCCCTAG...TAATTTCTATAGGT**GGGATTCACTGGGAAC TACG**AGGAATACTTTGGACTCGCAACTGATGTGGATGCTG...CAATTACCATTGGTGAAGATGTAAGTAG...

... AGACAACAAGGTGCCCTAG...TAATTTCTATAGGT**GGGATTCACTGGGAACTTACG**AGGAATACTTTGGACTCGCAACTGATGTGGATGCTG...CAATTACCATTGGTGAAGATGTAAGTAG...

WT

227-25

1 bp insertion

227-25:


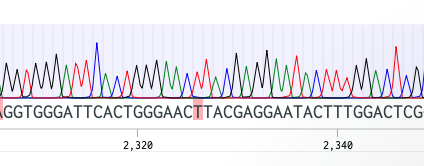


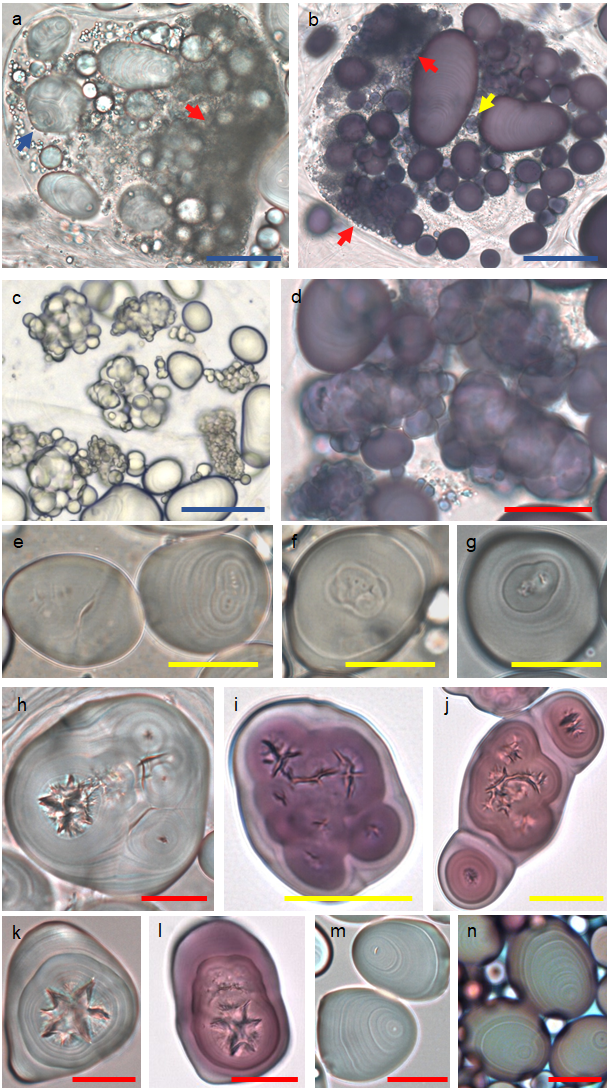


**Figure S15:** Images of tuber starch granules from mutant and wild-type lines.

**a**, **b**, Separated tuber cells of the mutant line 212-36. Examples of clusters of tiny granules are indicated with red arrows; nodular and multiple-hila granules are indicated with yellow and blue arrows respectively. **c** and **d**, Examples of nodular granules in purified starch from mutant 212-36 line without (**c**) and with (**d**) iodine staining. **e**, **f**, **g**, Granules with multiple, closely associated hila from mutant line 227-25 (**e**, **f**) and 237/4-44 (**g**). **h**, **i**, **j**, Granules with multiple hila from mutant line 237/4-44 in which separate granules with cracks across the hilum appear to have fused and then continued to grow as a single granule. **k**, **l**, Granules from mutant line 237/4-44 with typical deep cracks across the hilum. **m**, **n**, starch from control lines. In **b**, **d**, **i**, **j**, **l**, **n**, starch was lightly stained with iodine solution. Scale bars represent 50 µm (blue); 25 µm (red); 20 µm (yellow).


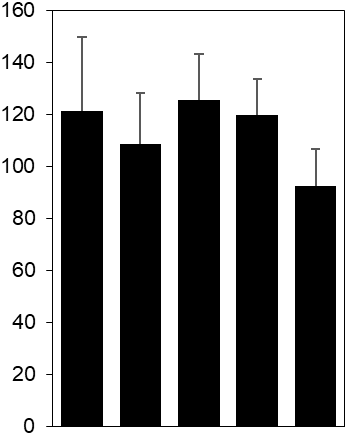


WT

230-51 (*sbe2*)

212-36 (*sbe1* and *2*)

227-25 (*sbe1* and *2*)

237/4-44 (*sbe1* and *2*)

Starch content (mg g^-1^ fresh weight)

**Figure S16.** Starch contents of mature tubers.

Samples were taken from the mid-cortex region at the midpoint of the tuber. Each sample was from a different tuber, and triplicate assays were performed on each sample in all but one case where duplicate assays were performed. Values are means ± SD of measurements on the following numbers of independent samples. WT, 3, from one plant; 230-51, 6, from 2 different plants; 212-36, 8, from 3 different plants; 227-25, 6, from 4 different plants; 237/4-44, 4, from one plant.

**Supplemental Experimental Procedures**

**HPLC-SEC**

Starch chain-length distributions were analysed following solubilisation and enzymatic debranching of purified starch using methods adapted from Perez-Moral et al. (2018). A Perkin-Elmer Series 200 HPLC (Llantrisant, UK) equipped with refractive index detector (RI), autosampler (35 °C), and column heater (80°C) was used for analysis of starches. Peak resolution by size exclusion was achieved using DMSO with 0.5% LiBr (w/v) at isocratic conditions with a 0.5 ml min^-1^ flow rate and a stationary phase involving two columns (8x300 mm, GRAM; Polymer Standard Service, Mainz, DE) in series (10 µm; 300 Å followed by 30 Å) and affixed to a guard column (8x50 mm, GRAM; Polymer Standard Service, Mainz, DE). Total run time was 60 min and injection volume for each sample was 50 μl, Calibration curves with correlation coefficients (R^2^ = 0.99996 ± 0.00003) were generated from pullulan standards (PSS-pulkit, Polymer Standard Service, Mainz, DE) having a range of peak molecular weights from 342 to 708,000 Da. Standards and starch samples were solubilized at 4 mg ml^-1^ for standards and 2 mg ml^-1^ for samples in DMSO containing 0.5% LiBr (w/v) and stored at 80°C overnight prior to analysis. A calibration curve was used to obtain a relationship between elution volume and hydrodynamic radius (V_h_) for the linear glucans as described by Cave et al., 2009. The RI elution profiles of the debranched starch samples were converted to SEC weight distributions as described in detail by Perez-Moral et al. (2018).

**^1^H NMR**

Samples were prepared for ^1^H NMR analysis of branch-point frequency as previously described (Schmitz et al., 2009; Tizzotti et al., 2011). The ^1^H NMR spectra were recorded at 600 MHz on a Bruker Avance HD spectrometer (Bruker BioSpin GmbH, Germany) running TopSpin 3.2 and fitted with a TCI cryoprobe. Spectra were acquired at 343°K using the zg30 pulse sequence with an acquisition time of 2.67 s, relaxation delay (D1) 12 s and 64 scans.
